# Developing a representative community health survey sampling frame using open-source remote satellite imagery in Mozambique

**DOI:** 10.1186/s12942-018-0158-4

**Published:** 2018-10-29

**Authors:** Bradley H. Wagenaar, Orvalho Augusto, Kristjana Ásbjörnsdóttir, Adam Akullian, Nelia Manaca, Falume Chale, Alberto Muanido, Alfredo Covele, Cathy Michel, Sarah Gimbel, Tyler Radford, Blake Girardot, Kenneth Sherr, with input from the INCOMAS Study Team, João Luis Manuel, Leecreesha Hicks, Arlete Mahumane, James Pfeiffer, Stephen Gloyd, Fatima Cuembelo, Miguel Nhumba, Joaquim Lequechane, Manuel Napua, Lucia Vieira

**Affiliations:** 1 Department of Global Health, University of Washington, 1959 NE Pacific Street, Seattle, WA 98195 USA; 2 Health Alliance International, Seattle, WA USA; 3 Universidade Eduardo Mondlane, Maputo, Mozambique; 4 Institute for Disease Modeling, Bellevue, WA USA; 5 Health Alliance International, Beira, Mozambique; 6 Beira Operations Research Center, Beira, Mozambique; 7 School of Nursing, University of Washington, Seattle, WA USA; 8 Humanitarian OpenStreetMap Team, Washington, DC USA

**Keywords:** Geographic information systems, Survey design, Satellite imagery, Remote sensing, Sampling, Evaluation, Mozambique

## Abstract

**Background:**

Lack of accurate data on the distribution of sub-national populations in low- and middle-income countries impairs planning, monitoring, and evaluation of interventions. Novel, low-cost methods to develop unbiased survey sampling frames at sub-national, sub-provincial, and even sub-district levels are urgently needed. This article details our experience using remote satellite imagery to develop a provincial-level representative community survey sampling frame to evaluate the effects of a 7-year health system intervention in Sofala Province, Mozambique.

**Methods:**

Mozambique’s most recent census was conducted in 2007, and no data are readily available to generate enumeration areas for representative health survey sampling frames. To remedy this, we partnered with the Humanitarian OpenStreetMap Team to digitize every building in Sofala and Manica provinces (685,189 Sofala; 925,713 Manica) using up-to-date remote satellite imagery, with final results deposited in the open-source OpenStreetMap database. We then created a probability proportional to size sampling frame by overlaying a grid of 2.106 km resolution (0.02 decimal degrees) across each province, and calculating the number of buildings within each grid square. Squares containing buildings were used as our primary sampling unit with replacement. Study teams navigated to the geographic center of each selected square using geographic positioning system coordinates, and then conducted a standard “random walk” procedure to select 20 households for each time a given square was selected. Based on sample size calculations, we targeted a minimum of 1500 households in each province. We selected 88 grids within each province to reach 1760 households, anticipating ongoing conflict and transport issues could preclude the inclusion of some clusters.

**Results:**

Civil conflict issues forced the exclusion of 8 of 31 subdistricts in Sofala and 15 of 39 subdistricts in Manica. Using Android tablets, Open Data Kit software, and a remote RedCap data capture system, our final sample included 1549 households in Sofala (4669 adults; 4766 children; 33 missing age) and 1538 households in Manica (4422 adults; 4898 children; 33 missing age).

**Conclusions:**

Other implementation or evaluation teams may consider employing similar methods to track population distributions for health systems planning or the development of representative sampling frames using remote satellite imagery.

**Electronic supplementary material:**

The online version of this article (10.1186/s12942-018-0158-4) contains supplementary material, which is available to authorized users.

## Background

The lack of updated and accurate estimates of population and their geographic distribution in low-and middle-income countries (LMICs) impairs planning, monitoring and evaluation of interventions, implementation of Ministry of Health (MOH) strategic plans, understanding health facility catchment areas critical for use with administrative data to calculate coverage estimates, and the accurate implementation of community-level surveys [1]. Like many LMIC countries, this is the case in Mozambique where civil registration is recognized to be suboptimal and important population displacement has been reported since the last population census conducted a decade ago in 2007 [2]. Existing census results are limited in spatial resolution to the provincial or district level (2nd and 3rd administrative levels), which hinders understanding the distribution of populations at the facility, subdistrict, or neighborhood level—levels at which health interventions are most often delivered.

Typically, generating a sampling frame for representative community sample surveys in LMICs requires manually enumerating target populations within the 12 months prior to the survey. This is the standard method employed by Demographic and Health Survey (DHS) [3] and Multiple Indicator Cluster Survey (MICS) [4] teams, and is logistically complex, requiring material resources, geographers, and engagement of local community structures to identify and enumerate large geographic areas. This is an expensive and difficult exercise that is not always feasible to perform. In addition, while this method has been seen as the “gold standard”, further efforts are needed to understand the validity and reliability of this method and how it may compare to alternative enumeration methods. For these reasons, fast and relatively inexpensive population enumeration and sampling methodologies are needed.

In the last decade, satellite data from Google Earth (a computer program owned by the Google Company that renders a 3D representation of the Earth based on satellite imagery and aerial photography) or other easily-accessible sources, have been used in a number of health-related applications in LMICs, including infectious disease surveillance [5–8], as an aid to the development of census mapping in Malawi [9], creating small-scale sampling frames for household surveys [10, 11], and a few examples of larger-scale sampling frames to estimate mortality in post-conflict Iraq [12] or to select households for specific health interventions [13]. In general, the use of satellite data to construct representative household survey sampling frames has been shown to be more rapid, less costly, and less biased compared to traditional sample enumeration methods [10, 12, 14, 15]. However, there are still relatively few practical guides on conducting large-scale sample frame enumeration (targeting an area covering 3–4 million inhabitants) using satellite imagery, and to our knowledge, no examples from sub-Saharan African contexts.

The purpose of this paper is to create and describe a method to allow the rapid enumeration and development of representative community sampling frames in LMICs using remote satellite imagery. Specifically, we describe the steps and procedures towards enumeration and sampling for a representative community survey in Manica and Sofala Provinces, Mozambique, as part of a program evaluation of a seven year health system strengthening intervention [16]. We hope to provide a practical explanation, including source programming code, to allow easy replication of our methods. Furthermore, the open-source nature of georeferenced building information from the OpenStreetMap project (www.openstreetmap.org) allows anyone to download the original georeferenced building data from Mozambique, or their own setting (if available), and apply the methods outlined herein.

## Methods

### Ethics approval

This study was approved by the Institutional Review Board of the National Institute of Health in Mozambique.

### Study setting

Mozambique is a southern East African country with some of the lowest rankings for health and development globally. The World Bank classifies Mozambique as a low-income country, with gross domestic product per capita in 2016 of US $1200 [17]. According to the 2015 Human Development Index, Mozambique ranks 181st out of 188 countries [18]. The country has 26.5 million inhabitants with more than half of the population under the age of 18, and 45% of the population under age 15. It also is among the eight countries with the highest HIV prevalence, with 13.2% of the adult population infected [19]. Mozambique has made great strides in decreasing under-5 and infant mortality over the past decade, although decreases have not been uniform across the country [20], with areas in the center and north of the country generally having higher mortality rates and lower statistics of development. Most (> 65%) of the population of Mozambique lives in rural areas, most often in groupings of households aggregated by a kinship system forming a homestead [21]. These homesteads are usually easy to identify as they are separated from other household groupings by significant distances or physical barriers such as fences or mud/concrete walls. Among countries in sub-Saharan Africa, only Swaziland, Lesotho, and Malawi have a higher percentage of their populations living in rural areas. Rural areas thus often have clusters of buildings surrounded by open areas of fields for subsistence farming—often each cluster of buildings represents a homestead with multiple generations cohabitating.

**Fig. 1:**
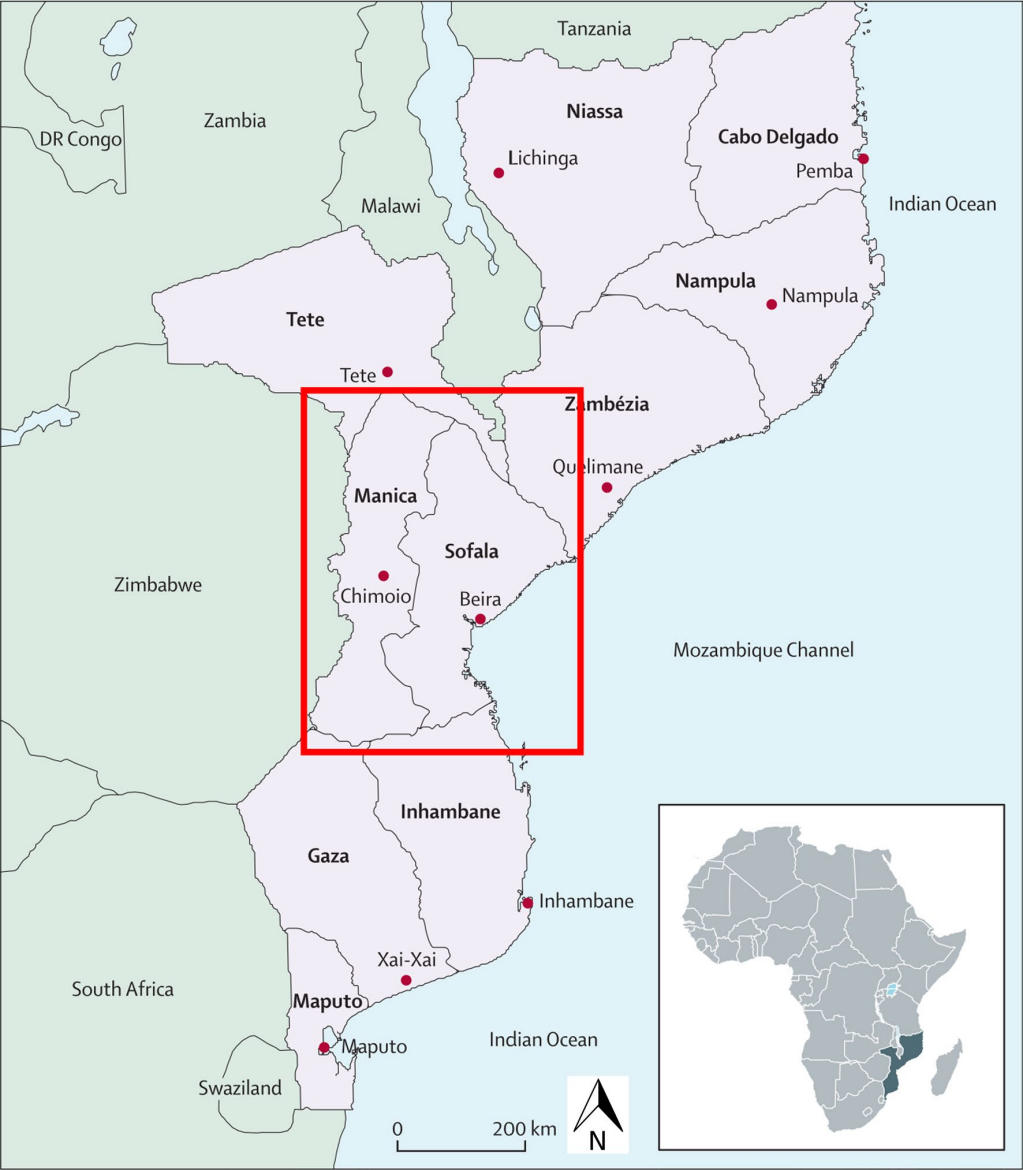
Political map of Mozambique, with red box outlining Sofala and Manica provinces, the target area for the present community survey sampling frame [20]

After gaining independence from Portugal in 1975, Mozambique endured a 16 year civil war, displacing millions and leading to near complete destruction of essential infrastructure. The civil war ended in 1992, with a period of stability and peace until 2012/2013, when an insurgency by the RENAMO political group (Mozambican National Resistance; Portuguese: Resistência Nacional Moçambicana) restarted, primarily affecting Sofala and Manica provinces in central Mozambique (see Fig. 1 for map of provinces). This insurgency against the country’s ruling FRELIMO (Mozambique Liberation Front; Portuguese: Frente de Libertação de Moçambique) has links to the decades-long Mozambican civil war and includes nighttime raids on cities and villages, as well as violent conflicts targeting main transport corridors. The fighting between RENAMO and FRELIMO intensified in 2014, leading to parts of Sofala and Manica provinces being unsafe to survey in the present investigation.

### Background on aim of survey and sample size calculations

The overall purpose of this study was to conduct a community survey to evaluate the impact of a 7-year health system strengthening intervention occurring in Sofala Province [16], using Manica Province as an evaluative control. In order to collect essential endline data for this impact evaluation, we conducted the following steps: (1) collaborative digitization and georeferencing of all visible buildings in Sofala and Manica Province using remote satellite imagery to serve as an estimate of population distribution for survey sampling; (2) development of probability proportional to size sampling frame using digitized buildings as a proxy for population distribution; (3) field implementation of house-to-house survey activities; (4) survey fidelity checks and the development of survey weights for data analysis. These steps are described in detail below, along with initial data on the performance of the field implementation procedures and the final sample drawn.

We based our sample size on the number of households visited for the standard DHS in Mozambique. The standard DHS in 2011 aimed to visit 1300 households in Sofala and 1200 in Manica [22]. To ensure our sample would exceed these numbers, we targeted a minimum of 1500 households in each province. Thus, we selected 88 grids within each province to reach 1760 households, anticipating ongoing conflict and transportation issues could preclude the inclusion of some clusters/households.

### Mapping buildings in Sofala and Manica provinces using satellite imagery

**Fig. 2:**
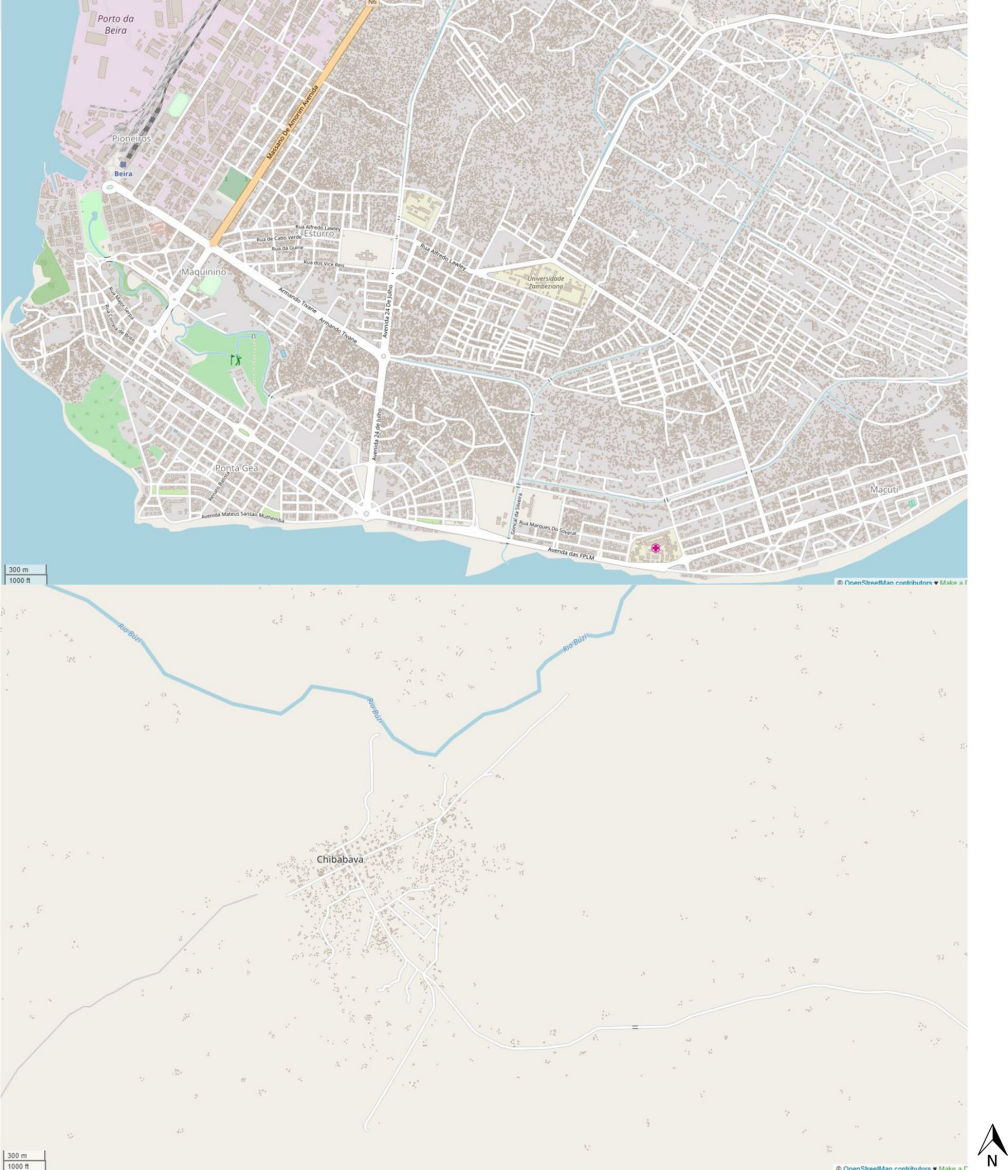
Two screenshots directly from OpenStreetMap showing formal and informal building settlements in Beira City (top screenshot, accessible: http://www.openstreetmap.org/#map=15/-19.8344/34.8557) and a more rural area of Chibabava (bottom screenshot, accessible: http://www.openstreetmap.org/#map=16/-20.2976/33.6509)

**Fig. 3:**
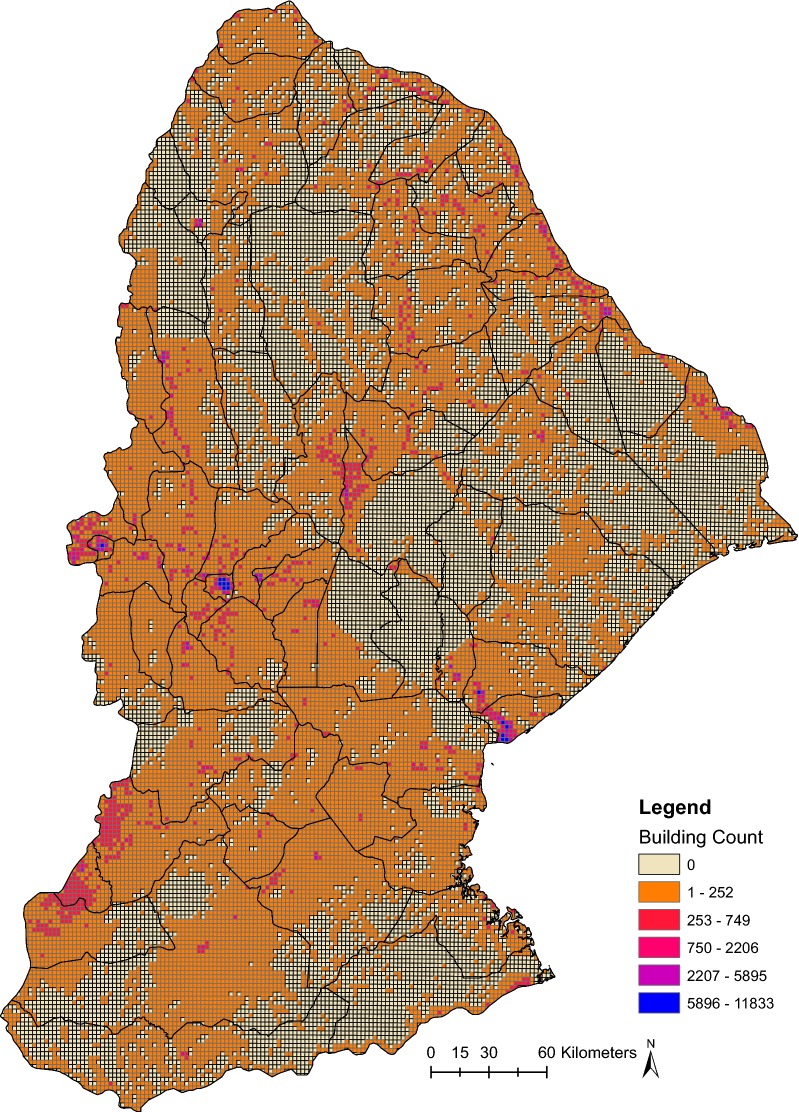
Sofala and Manica provinces with primary sampling unit (PSU) grid overlaid and cells colored by number of buildings contained within each PSU

**Fig. 4:**
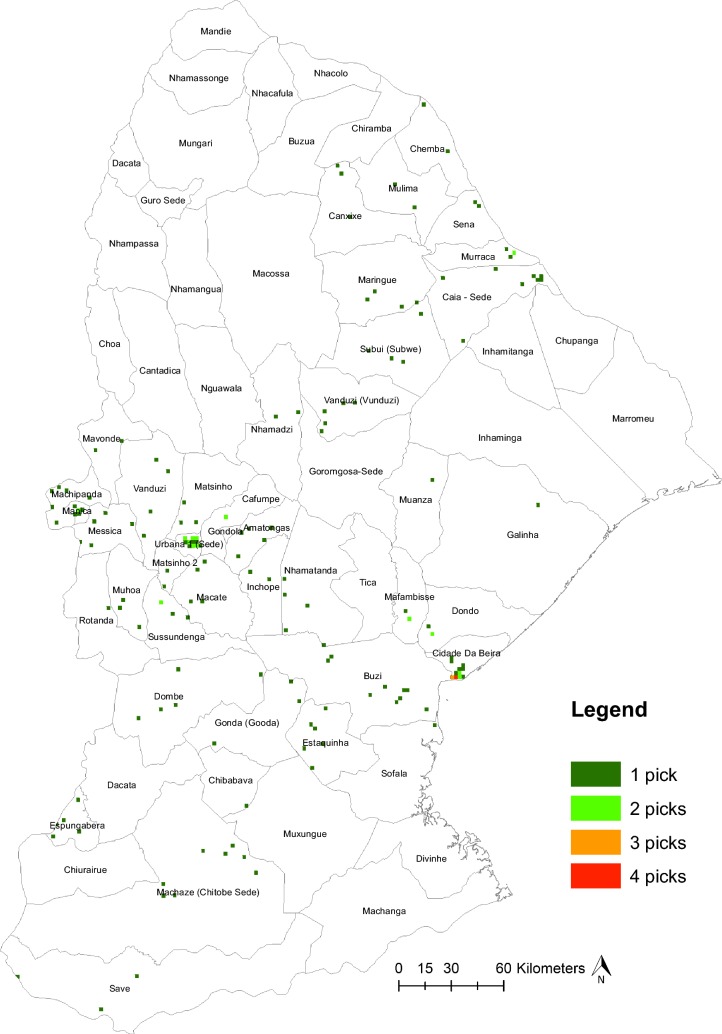
Selected grids within Provincial subdistricts after excluding conflict areas. Total sample is 157 boxes, 79 in Manica Province, 78 in Sofala Province. Total number of grids selected with replacement is 176 (88 per Province). Some grids are selected multiple times indicated by the color (dark green = 1 selection (20 households); neon green = 2 selections (40 households); orange = 3 selections (60 households); red = 4 selections (80 households)

**Fig. 5:**
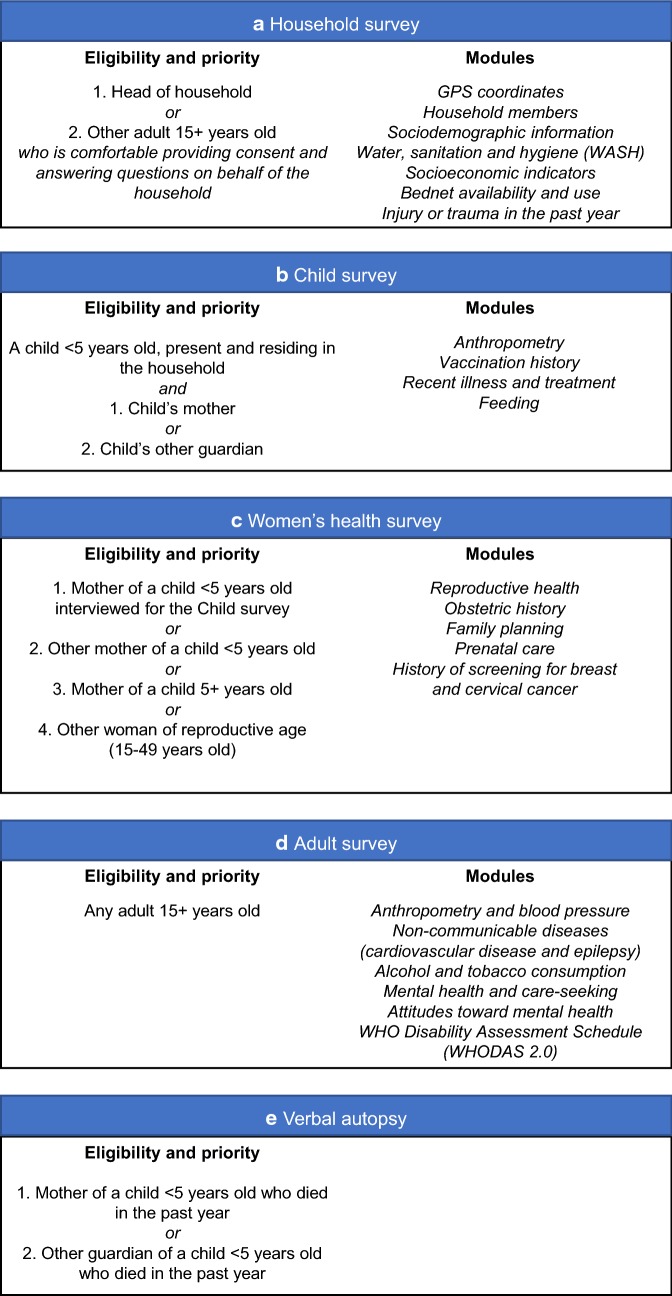
Description of key populations surveyed, eligibility and priority criteria, as well as survey modules applied for each population

Health Alliance International (HAI) contracted Humanitarian OpenStreetMap Team (HOT), a Non-governmental organization (NGO) and global mapping community, to digitize and georeference all visible buildings in Sofala and Manica Provinces to serve as an estimate of population distribution for survey sampling. The HOT team worked with a team of 20 trained mappers out of Dar Es Salaam, Tanzania who utilized Java OpenStreetMap Editor to trace polygons on all buildings in the two provinces visible by satellite imagery. Each mapper traced polygons in a given work area, which was then reviewed by an expert quality-control supervisor prior to confirmation and being uploaded into the OpenStreetMap database. These mapping activities started in February 2015 and were completed in May 2015, with a total of 1,610,902 building digitized (685,189 Sofala; 925,713 Manica). Basemap satellite imagery was obtained from Microsoft Bing and Mapbox Satellite, with all basemap imagery being from 2013 to 2016. Interested parties can view the final mapped buildings at www.openstreetmap.org, and export the most up-to-date most up-to-date OpenStreetMap project data for Mozambique at: https://download.geofabrik.de/africa/mozambique.html. Also, see Fig. 2 for examples of digitized building polygons in Beira City, Sofala, Mozambique, as well as a more rural area of Chibabava district, Sofala, Mozambique. Customized thematic maps for Figs. 3, 4 and 5 were created in ArcMap 10.5.

### Developing primary sampling units for probability proportional to size sampling

Once the building digitization was complete, we exported the shapefiles—including building shapes for Sofala and Manica—from the OpenStreetMap platform. We then used R 3.2.3 (Comprehensive R Archive Network) to overlay a grid on Sofala and Manica Provinces using the GridFilter command explained at http://bit.ly/2xCI5Ge (see Additional file 1 for raw R code). The spatial resolution we chose for the primary sampling unit “grid” (PSU) was 0.02 decimal degrees, with each box being 2.106 km across using the Universal Transverse Mercator Projected Coordinate System 37S (UTM PCS 37S) for Mozambique. This resolution balanced the inherent tension between granularity of the sampling frame and attempting to minimize the number of grid PSUs that would contain no buildings or a number of buildings less than 20, which was the number to be selected from each PSU. We then tabulated the number of buildings within each PSU and exported the final database file including each PSU paired with a building count using R. This resulted in 28,391 total PSUs (14,764 Sofala; 13,627 Manica). Even with this resolution, this resulted in 12,178 PSUs (7004 Sofala; 5174 Manica) with no buildings. Thus, the final PSU numbers for probability proportional to size (PPS) sampling were 16,213 (7760 Sofala; 8453 Manica). PPS sampling here means that the probability of selecting each PSU “grid” cell was directly proportional to the number of buildings contained within each PSU “grid” cell. Of these PSUs included in the final sample, the mean building count was 99.3 (SD = 341.9), with a median building count of 44. See Fig. 3 for a thematic heatmap of these PSUs colored by the number of buildings in each 2.1 × 2.1 km grid cell.

### Carrying out probability proportional to size sampling

Due to ongoing violent civil conflict, a number of subdistricts in both Sofala and Manica Provinces were unsafe for travel or survey research in September, 2016 when activities were to be launched. This forced our team to exclude all PSUs lying within the subdistricts of Marromeu, Chupanga, Inhamitanga, Inhaminga, Gorongosa-Sede, Mixungue, Divinhe, and Machanga in Sofala and Mandie, Nhamassonge, Nhacafula, Nhacolo, Mungari, Buzua, Dacata, Guro-Sede, Macossa, Nhamangua, Nhampassa, Choa, Catadica, Nguawala, and Chiurairue in Manica prior to drawing our final PPS sample (see Fig. 4 for the locations of these excluded subdistricts). This was unfortunate but necessary to maintain the safety of our research teams. Survey implementation could not be delayed due to the need to measure key indicators as close to the end of our intervention as possible. Intervention activities ended in September 2015, and thus our survey already had a year’s delay between survey initiation and the end of intervention activities.

We then used the SamplePPS command in Stata 14 (available from the Boston College Statistical Software Components archive) which draws a random sample from a current dataset with probabilities proportional to size. In our case, the PSUs were our aforementioned grid, and our size was the number of buildings in each grid. See Additional file 2 for detailed Stata 14 code for PPS sampling. We sampled 88 PSUs within each Province with replacement. A number of the highest density PSUs were sampled multiple times, such as those in Beira and Chimoio cities (see Fig. 4).

### Field implementation

Prior to initiating field procedures, each supervisor entered the Global Position System (GPS) location of the center of each PSU their team was responsible for into OpenStreetMap or Google Maps and determined the optimal transport route to this point. The center of each PSU was calculated using ArcMap 10.5. Many of our sampled PSUs were in very rural areas of central Mozambique with limited to no paved roads—many of which are inaccessible many months of the year due to rains, failed bridges, or other infrastructural challenges. Thus, supervisors had detailed discussions with HAI expert staff drivers to determine individual logistics plans to visit each PSU.

In OpenStreetMap, the supervisor also noted the location of the households closest to the central GPS point. Once arriving at a given PSU, field teams used GPS location on tablet-based Android survey devices and the GPS navigation application Sygic to identify the geographic center of the PSU. Sygic is a tablet or phone-based navigation software that can operate without an active internet connection (more information: https://www.sygic.com/gps-navigation). In rural areas the closest houses to this central GPS location were sampled first, with subsequent households being those whose front door was closest to the front door of the initial household sampled following methods outlined in the World Health Organization’s (WHO) Expanded Programme on Immunization methods [23]. In urban areas, or areas where there were multiple houses equidistant from the starting center GPS location, these households were numbered, with one household randomly selected as the starting household. If the GPS location for the center of the PSU was in a creek, or a lake, supervisors observed this by plotting in OpenStreetMap prior to initiating field activities, and visually identified the household closest to this GPS location to serve as the starting sampling unit. If the closest building to the center of a given PSU was a commercial building, field teams excluded this building from consideration and traveled to the closest residential building. If an apartment complex was selected, the research teams randomly sampled one household every two floors.

Approximately 1 week prior to visiting PSUs, research teams contacted community leaders to notify the population that a survey would be undertaken. When research teams arrived at PSUs, they first visited these community leaders, who often traveled with the teams to households sampled—this led to near universal consent participate in the survey and helped ensure that sampled participants were home and prepared to answer survey questions.

When arriving at a household, research teams first asked to talk with the self-appointed “head of household”. This individual was asked to conduct initial informed consent and answer questions related to the general household questionnaire (household demographics, assets, etc.). If the “head of household” was not present, the teams asked to interview another adult ≥ 15 who was comfortable answering questions on behalf of the household. After completing the household survey, this individual completed the full adult questionnaire. All individuals surveyed were administered questionnaires in a private area of their homestead. After surveying the “head of household” or household proxy, research teams were instructed to select a woman with a child < 5 who were both present in the household and administer the Child and Women’s Health surveys, including anthropometry and questions on birth history and maternal/child health. If no such woman was available, research teams were to administer the Women’s Health survey to another mother of a child < 5; if no mother of a child < 5 was available, this survey was administered to a mother of a child ≥ 5; and if there we no mothers present, they were to administer the Women’s Health survey to another woman of reproductive age (15–49 years old). If there were no further women of reproductive age in the household, research teams were to administer the Adult survey to any other adults ≥ 15 in the household attempting to maximize gender diversity. That is, if the teams had interviewed two women of reproductive age they would interview a male adult. If the teams had interviewed a man as “head of household” and a woman of reproductive age and there were both men and women not 15–49 years old, they would randomly select the next interviewee. A maximum of three individuals were interviewed per household. If no one was present at the sampled household, teams would move to the next household in the sampling procedure. Field implementation progressed in three research teams, each with 3–5 research assistants, one supervisor, and one car. A figure explaining sampling populations, sampling criteria, and survey modules applied to each population group can be seen in Fig. 5.

### Data management and collection

Data were collected on Samsung tablets using Open Data Kit (ODK) software (https://opendatakit.org/). Data were directly transferred from ODK to a REDCap database [24] through a cloud server in real time. Household questionnaires were adapted from the Mozambique DHS with additional modules to estimate the burden of cardiovascular disease, mental health conditions, alcohol abuse and epilepsy, as well as a general disability module (see Additional file 3 for full survey materials).

### Sampling weight calculation

To calculate sampling weights, we calculated both the probability of selecting each PSU (Eq. 1 below) and the probability of household selection within each PSU (Eq. 2 below).

The probability of each cluster *i* being sampled in each province *h* is given by:

1 $$P_{1ih} = \frac{{\# buildings_{i} \times \# clusters\;to\;be\;sampled_{h} }}{{\# total\;buidings_{h} }}$$

The probability of each household *j* being sampled in each cluster *i* is given by:

2 $$P_{2ji} = \frac{\# households\;to\;select\;per\;cluster}{{\# buildings_{i} }}$$

If the number of buildings in a PSU was bigger than sampling interval, the probability to select the PSU was set to 1. Additionally, if the number of buildings in a PSU was less than 20, the probability to select households was also set to 1. The overall basic weight of household sampling was the inverse of the probability of selection (Eq. 3 below).

Overall sampling weight of the household is given by:

3 $$W_{j} = \frac{1}{{\left( {P_{1ih} *P_{2ji} } \right) }}$$

Out of 176 PSUs (88 PSUs per province), 23 PSUs were excluded because of ongoing regional conflict. As non-response adjustment, the sampling weights of these 23 PSUs were redistributed to other PSUs at the Provincial level, consistent with the stratification of PSUs at the Provincial level.

## Results of field implementation

As a result of PPS sampling, 78 unique grid cells (PSUs) were selected to be sampled in Sofala, with 79 selected to be sampled in Manica. In Sofala, 71 PSUs were selected once, 5 PSUs were selected twice, one 3 times, and one 4 times. In Manica, 70 PSUs were selected once, and 9 were selected 2 times (see Fig. 4). Unfortunately, even after excluding a number of sub-districts prior to drawing the PPS sample, during field implementation we were unable to visit 12 PSUs in Sofala and 11 PSUs in Manica due to ongoing civil conflict restricting travel (see Fig. 5). All PSUs research teams were unable to visit were sampled only once.

Survey field implementation ran from September 29th, 2016 to February 18th, 2017. The final sample after face-to-face interviews and field implementation included 3087 total households, with 1549 in Sofala and 1538 in Manica. These households included 4669 adults sampled, 4766 children sampled, and 33 missing age in Sofala and 4422 adults sampled, 4898 children sampled, and 33 missing age in Manica. Survey teams only recorded one instance of survey refusal.

## Discussion

The present study outlines, in a practical fashion, how we generated a population representative sampling frame for two provinces in central Mozambique when up-to-date census data were not available. We used satellite imagery integrated with the open-source OpenStreetMap platform to digitize all buildings, which were then used to represent population density and generate a PPS sample. Not only was this approach feasible, low-cost, and rapid for our group, the integration with the open-source OpenSteetMap platform means that anyone can utilize our digitized basemap information for any purpose, whether for generating another sampling frame, or for health system planning and implementation. We suggest that similar approaches using satellite imagery to generate sampling frames integrate as much as possible with open-source mapping databases, such as OpenStreetMap, to avoid unnecessary duplication of geocoding and mapping activities which can be time consuming, laborious, and may be cost-prohibitive for many entities in LMICs.

Our approach has a number of important limitations. First, our sampling frame relied on strict building counts within grid cells, which fails to account for the size of a building, or the number of occupants in a given building. This may have had the effect of under-sampling urban areas which tend to have larger buildings, such as apartment complexes, housing large numbers of individuals and multiple households. Second, although all efforts were made to develop a representative sampling frame, we were forced to exclude large areas of Sofala and Manica provinces due to ongoing violent civil conflict. Even after our sample was drawn, we were unable to visit all PSUs due to unforeseen shifts in fighting. This is a regrettable outcome that will likely bias our final survey results. Last, for simplicity in field implementation, we relied on the admittedly outdated WHO “random walk” procedure to select households randomly within each PSU. Other methods have been developed for this stage of sampling, including randomly selecting a starting grid or randomly selecting a starting household within each PSU. However, these can have additional implementation challenges, and some implementers have found the WHO “random walk” procedure to result in findings that are not significantly biased compared to these more complicated procedures [25].

Our approach also has a number of strengths. Instead of relying on “black box” population modelling data from groups such as WorldPop (http://www.worldpop.org.uk/), we relied on up-to-date satellite imagery and a straightforward approach of building count within each grid cell to represent the distribution of population. This included enumerating a census of all buildings in Sofala and Manica provinces, so the approach was consistently applied across all areas. Furthermore, we integrated the effort to digitize buildings with the open-source OpenStreetMap platform, which allows anyone to utilize these data for other purposes. For example, if one could accurately estimate the number of individuals living within each building based on building characteristics, one could directly estimate population to a neighborhood level to improve program planning from the MOH, and potentially to generate more accurate population catchment areas for facility-level administrative data.

### Future directions

Future studies could assess the extent to which building counts alone correlate with population density and population distribution over large areas, such as provinces or districts in LMICs. This was difficult to do in our setting as we do not have available or up-to-date “gold standard” census data to validate or compare with building count population estimates at the sub-district resolution used for sampling in this study. In addition, a specific future study could conduct a household-level census and compare to building counts over diverse geographic areas. This would help understand the extent to which building counts alone can be used as a proxy for population distributions. Other studies should also be conducted comparing PSU selection methods that balance complexity and validity, including the potential development of new methods that are easy to implement but valid. Last, future studies could compare the validity of sampling frames developed using gold-standard household census data to those using building or other satellite-imagery data and/or gridded population estimates such as WorldPop (http://www.worldpop.org.uk/) that provide estimates of populations at the 100 × 100 m grid cell for every LMIC globally.

## Conclusions

We hope the practical guide outlined here, including supplementary R and Stata code, along with integration with open-source platforms can be useful to other groups seeking to replicate our approach, or build and improve upon it. Based on our experience, we suggest that groups needing population-representative sampling frames consider using satellite imagery instead of more laborious field enumeration, which is often not feasible for larger-scale surveys. In addition, innovative methods for using georeferenced satellite data to inform real-time MOH program planning, health systems organization and planning, understanding of rapid population movements, and developing accurate catchment areas to use facility-level administrative data for coverage estimates are needed. Integrating these efforts with open-source platforms such as OpenStreetMap, will allow efficient and rapid innovation to drive equitable health systems and outcomes globally.

## Additional files

**Additional file 1.** R code used to overlay sampling grid, generate building counts within grid cells, and output shapefile.

**Additional file 2.** Stata code used to generate probability proportional to building count sample.

**Additional file 3.** Full survey instrument used in Sofala and Manica, Mozambique. This survey was designed and implemented in Portuguese, and is provided here translated into English. Household questionnaire is listed first, followed by the individual questionnaire.

## Abbreviations

LMICs: low-and middle-income countries
MOH: Ministry of Health
DHS: Demographic and Health Survey
MICS: Multiple Indicator Cluster Survey
IMASIDA: national community survey on immunization, malaria, and HIV/AIDS
HAI: Health Alliance International
HOT: Humanitarian OpenStreetMap Team
NGO: non-governmental organization
PSU: primary sampling unit
PPS: probability proportional to size
GPS: global positioning system
WHO: World Health Organization
ODK: Open Data Kit

## References

[1] Viel J, Tran A (2009). Estimating denominators: satellite-based population estimates at a fine spatial resolution in a european urban area. Epidemiology.

[2] Ye Y, Wamukoya M, Ezeh A, Emina JBO, Sankoh O (2012). Health and demographic surveillance systems: a step towards full civil registration and vital statistics system in sub-Sahara Africa ?. BMC Public Health.

[3] ICF International (2012). Sampling and household listing manual: demographic and health surveys methodology.

[4] UNICEF. Multiple indicator cluster survey manual: chapter 4 designing and selecting the sample. 2017.

[5] Chang AY, Parrales ME, Jimenez J, Sobieszczyk ME, Hammer SM, Copenhaver DJ (2009). Combining Google Earth and GIS mapping technologies in a dengue surveillance system for developing countries. Int J Health Geogr..

[6] Gammino VM, Nuhu A, Chenoweth P, Manneh F, Young RR, Sugerman DE (2014). Using geographic information systems to track polio vaccination team performance: pilot project report. J Infect Dis.

[7] Kabaria CW, Molteni F, Mandike R, Chacky F, Noor AM, Snow RW (2016). Mapping intra-urban malaria risk using high resolution satellite imagery: a case study of Dar es Salaam. Int J Health Geogr..

[8] Rogers DJ, Randolph SE, Snow RW, Hay SI (2002). Satellite imagery in the study and forecast of malaria. Nature.

[9] Chirwa I. Census mapping using satellite imagery in Malawi. In: United Nations Regional Workshop on the 2020 World Programme on Population and Housing Censuses. Lusaka, Zambia; 2017. https://www.goog. Accessed 26 Sept 2017.

[10] Escamilla V, Emch M, Dandalo L, Miller WC, Hoffman I (2014). Sampling at community level by using satellite imagery and geographical analysis. Bull World Health Organ.

[11] Wampler P, Rediske R, Molla A (2013). Using ArcMap, Google Earth, and Global Positioning Systems to select and locate random households in rural Haiti Using ArcMap, Google Earth, and Global Positioning Systems to select and locate random households in rural Haiti. Int J Health Geogr..

[12] Galway LP, Bell N, Ae S, Shatari A, Hagopian A, Burnham G (2012). A two-stage cluster sampling method using gridded population data, a GIS, and Google Earth TM imagery in a population-based mortality survey in Iraq. Int J Health Geogr..

[13] Kamanga A, Renn S, Pollard D, Bridges DJ, Chirwa B, Pinchoff J (2015). Open-source satellite enumeration to map households: planning and targeting indoor residual spraying for malaria. Malar J..

[14] Shannon HS, Hutson R, Kolbe A, Stringer B, Haines T (2012). Choosing a survey sample when data on the population are limited: a method using Global Positioning Systems and aerial and satellite photographs. Emerg Themes Epidemiol..

[15] Haenssgen MJ (2015). Satellite-aided survey sampling and implementation in low- and middle-income contexts: a low-cost/low-tech alternative. Emerg Themes Epidemiol..

[16] Sherr K, Cuembelo F, Michel C, Gimbel S, Micek M, Kariaganis M (2013). Strengthening integrated primary health care in Sofala, Mozambique. BMC Health Serv Res..

[17] United States Central Intelligence Agency. CIA World Factbook: Mozambique. https://www.cia.gov/library/publications/the-world-factbook/geos/print_mz.html. Accessed 27 Sept 2017.

[18] United Nations Development Programme. Human Development Report 2016: Mozambique. http://hdr.undp.org/sites/all/themes/hdr_theme/country-notes/MOZ.pdf. Accessed 27 Sept 2017.

[19] Mozambican Ministry of Health. Inquérito de Indicadores de Imunização, Malária e HIV/SIDA em Moçambique (IMASIDA). 2016.

[20] Fernandes QF, Wagenaar BH, Anselmi L, Pfeiffer J, Gloyd S, Sherr K (2014). Effects of health-system strengthening on under-5, infant, and neonatal mortality: 11-year provincial-level time-series analyses in Mozambique. Lancet Glob Heal..

[21] Davila J, Kyrou E, Nunez T, Sumich J (2008). Urbanisation and municipal development in Mozambique: urban poverty and rural–urban linkages.

[22] Instituto Nacional de Estatística (2013). Inquérito Demográfico e de Saúde 2011—Moçambique.

[23] World Health Organization (2008). Training for mid-level managers (MLM): the EPI coverage survey.

[24] Harris PA, Taylor R, Thielke R, Payne J, Gonzalez N, Conde JG (2009). Research electronic data capture (REDCap)—a metadata-driven methodology and workflow process for providing translational research informatics support. J Biomed Inform.

[25] Grais RF, Rose AMC, Guthmann J (2007). Don’t spin the pen: two alternative methods for second-stage sampling in urban cluster surveys. Emerg Themes Epidemiol.

