# Supplementary material for: Developing a representative community health survey sampling frame using open-source remote satellite imagery in Mozambique
Source: Int J Health Geogr. 2018 Oct 29;17:37. doi: 10.1186/s12942-018-0158-4 (PMC6206736; doi:10.1186/s12942-018-0158-4)
Supplement: Supplementary file 1 — Additional file 1. R code used to overlay sampling grid, generate building counts within grid cells, and output shapefile. [file 12942_2018_158_MOESM1_ESM.pdf]

Additional File 1. R code used to overlay sampling grid, generate building counts within grid cells, and output shapefile.

```
##################################################################################################################
# Mozambique Sampling Frame for Doris Duke Evaluation
# June 21, 2016
# ###############################################################################################################

.libPaths("C:/USER") #folder for libraries
library("colorspace")
library("xts")
library("intervals")
library("stringi")
library("xts")
library("GenKern")
library("ggplot2")
library("plyr")
library("ggplot2")
library("prevR")
library("maptools")
library("readstata13")
library("rgeos")
library("sp")
library("gam")
library("MapGAM")
library("knitr")
library("foreach")
library("PBSmapping")
library("mgcv")
library("splancs")
library("akima")
library("gstat")
library("spacetime")
library("raster")
library("MASS")

setwd("C:/USER ")
root <- "C:/USER"

admin0 <- readShapeSpatial("Admin shapes/MOZ_adm0.shp")
admin1 <- readShapeSpatial("Admin shapes/MOZ_adm1.shp")
admin2 <- readShapeSpatial("Admin shapes/MOZ_adm2.shp")
admin3 <- readShapeSpatial("Admin shapes/MOZ_adm3.shp")
ms.shape <- readShapeSpatial("Admin shapes/ms_admin1_2")

#Create a grid over the study region
admin0 <- readShapeSpatial("Admin shapes/MOZ_adm0.shp")
admin1 <- readShapeSpatial("Admin shapes/MOZ_adm1.shp")
admin2 <- readShapeSpatial("Admin shapes/MOZ_adm2.shp")
admin3 <- readOGR("Admin shapes", "MOZ_adm3")
shape <- readOGR("Admin shapes", "DissolvedAdm1")

plot(admin0)
plot(admin1, add=T)
plot(admin2, add=T)
unique(admin1$NAME_1)

plot(ms.admin1)
s.buildings <- read.csv("buildings/sofalaxyarea.csv")
m.buildings <- read.csv("buildings/manicaxyarea.csv")
head(s.buildings)
head(m.buildings)
sm.buildings <- rbind(s.buildings, m.buildings)
head(sm.buildings)

sm.buildings.p <- as.points(sm.buildings$x, sm.buildings$y)
#plot(sm.buildings.p, pch=19, col="blue")

#Create a grid over the study region

GridFilter<-function(shape, resol = 1, prop = 0){  #This function from http://rfunctions.blogspot.com/2014/12/gridfilter-intersect-grid-with-shape.html
  grid <- raster(extent(shape))
  res(grid) <- resol
```

```
  proj4string(grid)<-proj4string(shape)
  gridpolygon <- rasterToPolygons(grid)
  drylandproj<-spTransform(shape, CRS("+proj=laea"))
  gridpolproj<-spTransform(gridpolygon, CRS("+proj=laea"))
  gridpolproj$layer <- c(1:length(gridpolproj$layer))
  areagrid <- gArea(gridpolproj, byid=T)
  dry.grid <- intersect(drylandproj, gridpolproj)
  areadrygrid <- gArea(dry.grid, byid=T)
  info <- cbind(dry.grid$layer, areagrid[dry.grid$layer], areadrygrid)
  dry.grid$layer<-info[,3]/info[,2]
  dry.grid <- spTransform(dry.grid, CRS(proj4string(shape)))
  dry.grid.filtered <- dry.grid[dry.grid$layer >= prop,]}

region.grid <- GridFilter(shape, resol=0.01, prop=0.1)  #set parameters, prop = proportion of grid covering shape to be included
#Note 0.01 decimal degrees = about 1km
plot(region.grid)

#plot(sm.buildings.p, col="blue", pch=19)
plot(region.grid)
plot(admin3, add=T)

sp = SpatialPoints(sm.buildings.p)
proj4string(sp)<-proj4string(region.grid)
region.grid$name <- as.numeric(row.names(region.grid))

# region.grid.o <- over(region.grid, admin3)
# region.grid.o$name <- as.factor(row.names(region.grid.o))
# region.grid.m <- merge(region.grid, region.grid.o)

res <- over(sp, region.grid)
res.s <- res[order(res$name),]

freq <- as.data.frame(tabulate(res$name)) #tabulate includes zero values (like for cell #40)
colnames(freq)="building.count"
freq$name <- row.names(freq)
region.grid.m.f <- merge(region.grid, freq, by="name")
region.grid.m.f$id <- region.grid.m.f$name
region.grid.m.f.gg <-fortify(region.grid.m.f, region="name")
region.grid.m.f.gg.m <- merge(region.grid.m.f.gg, as.data.frame(region.grid.m.f), by="id")

values=c(0,60000)
limits=c(0,1)
breaks = c(0,10000,30000, 40000,60000)

ggplot(data = subset(region.grid.m.f.gg.m, building.count!=0), aes(x = long, y = lat, fill = building.count, group = group)) +
  geom_polygon() +
  scale_fill_gradientn(name="Proportion",
      colours= c("darkgreen","yellow","red","darkred"),
      breaks = breaks,
      na.value = NA) +
  coord_equal() +
  theme()

writeOGR(region.grid.m.f, "C:/USER", "buildings.scl_0.1", driver="ESRI Shapefile", overwrite_layer=T)
```
