# Supplementary material for: Developing a representative community health survey sampling frame using open-source remote satellite imagery in Mozambique
Source: Int J Health Geogr. 2018 Oct 29;17:37. doi: 10.1186/s12942-018-0158-4 (PMC6206736; doi:10.1186/s12942-018-0158-4)
Supplement: Supplementary file 2 — Additional file 2. Stata code used to generate probability proportional to building count sample. [file 12942_2018_158_MOESM2_ESM.pdf]

Additional File 2. Stata code used to generate probability proportional to building count sample.

```
*Import grid with districts merged
import delimited "C:\USER", varnames(1)

*install samplepps program
ssc install samplepps

* SELECT SAMPLE (3500 total sample; 20 per box; equal boxes Sofala Manica)
*(Thus, 88 per province, yielding 176 boxes)

*Select sample excluding conflict zones
*Seed from RANDBETWEEN function in Excel
set seed 256988892
samplepps picksofala if Sofala==1 & conflict==0, size(buildingsperarea) n(88) withrepl

samplepps pickmanica if Sofala==0 & conflict==0, size(buildingsperarea) n(88) withrepl

*export 188 box sample
export delimited using "C:\USER", replace
```
