# Supplementary material for: Developing a representative community health survey sampling frame using open-source remote satellite imagery in Mozambique
Source: Int J Health Geogr. 2018 Oct 29;17:37. doi: 10.1186/s12942-018-0158-4 (PMC6206736; doi:10.1186/s12942-018-0158-4)
Supplement: Supplementary file 3 — Additional file 3. Full survey instrument used in Sofala and Manica, Mozambique. This survey was designed and implemented in Portuguese, and is provided here translated into English. Household questionnaire is listed first, followed by the individual questionnaire. [file 12942_2018_158_MOESM3_ESM.pdf]

MANICA AND SOFALA COMMUNITY
SUREY (InCoMaS)
HOUSEHOLD SURVEY

CONFIDENTIAL

IDENTIFICATION

GPS COORDINATES
Latitude
Longitude
HOUSEHOLD'S STUDY ID
PROVINCE (1=SOFALA 2=MANICA)
URBAN / RURAL (URBAN=1, RURAL=2)

NUMBER OF PEOPLE IN THE HOUSEHOLD
NUMBER OF CHILDREN AGED 0-4
NUMBER OF CHILDREN AGED 5-14
NUMBER OF WOMEN 15+
NUMBER OF MEN 15+

HOUSEHOLD SELECTED FOR CHILDREN'S SURVEY? (1=YES, 2=NO) . . . .

HOUSEHOLD SELECTED FOR WOMEN'S SURVEY? (1=YES, 2=NO) . . . .

HOUSEHOLD SELECTED FOR MEN'S SURVEY . . . .

INTERVIEWER VISITS

	1	2	3	FINAL VISIT
DATE	________	________	________	DAY<br>MONTH<br>YEAR
INTERVIEWER'S NAME	________	________	________	INT. NO.
RESULT*	________	________	________	RESULT*
NEXT VISIT: DATE<br>TIME	________<br>________	________<br>________		TOTAL NUMBER OF VISITS

*RESULT CODES:

1 COMPLETED
2 NO HOUSEHOLD MEMBER AT HOME OR NO COMPETENT RESPONDENT AT HOME AT TIME OF VISIT
3 ENTIRE HOUSEHOLD ABSENT FOR EXTENDED PERIOD OF TIME
4 POSTPONED
5 REFUSED
6 DWELLING VACANT OR ADDRESS NOT A DWELLING
7 DWELLING DESTROYED
8 DWELLING NOT FOUND
9 OTHER ______________________________
(SPECIFY)

____

INTERVIEWER

NAME

Eligibility

Please tell me if there are people who habitually live in this houshold from the following categories:

**Maternal child health**	
Children between 0-4 years of age	A
Children between 5-14 years of age	B
Mothers of children 14 and under	C
Other women of childbearing age, 15-49 years of age	D
**Non-transmissible diseases and cardiovascular risk**	
Men 15 years old and older	E
Women 50 years old and older	F

LINE NO.	USUAL RESIDENTS AND VISITORS	RELATIONSHIP TO HEAD OF HOUSEHOLD	SEX	RESIDENCE		AGE	IF AGE 15 OR OLDER<br>MARITAL STATUS	ELIGIBILITY		
1	2	3	4	5	6	7	8	9	10	11
	Please give me the names of the persons who usually live in your household and guests of the household who stayed here last night, starting with the head of the household.<br><br>AFTER LISTING THE NAMES AND RECORDING THE RELATIONSHIP AND SEX FOR EACH PERSON, ASK QUESTIONS 2A-2C TO BE SURE THAT THE LISTING IS COMPLETE.<br><br>THEN ASK APPROPRIATE QUESTIONS IN COLUMNS 5-20 FOR EACH PERSON.	What is the relationship of (NAME) to the head of the household?<br><br>SEE CODES BELOW.	Is (NAME) male or female?	Does (NAME) usually live here?	Did (NAME) stay here last night?	How old is (NAME)?<br><br>IF 95 OR MORE, RECORD '95'.	What is (NAME)'s current marital status?<br><br>1 = MARRIED OR LIVING TOGETHER<br>2 = DIVORCED/ SEPARATED<br>3 = WIDOWED<br>4 = NEVER-MARRIED AND NEVER LIVED TOGETHER	CIRCLE LINE NUMBER OF ALL WOMEN AGE 15-49	**IF HOUSE-HOLD SELEC-TED FOR MAN'S SURVEY**<br><br>CIRCLE LINE NUMBER OF ALL MEN AGE 15-[49]	CIRCLE LINE NUMBER OF ALL CHILDREN AGE 0-5
			M F	Y N	Y N	IN YEARS				
01		☐☐	1 2	1 2	1 2	☐☐	☐	01	01	01
02		☐☐	1 2	1 2	1 2	☐☐	☐	02	02	02
03		☐☐	1 2	1 2	1 2	☐☐	☐	03	03	03
04		☐☐	1 2	1 2	1 2	☐☐	☐	04	04	04

2A) Just to make sure that I have a complete listing: are there any other people such as small children or infants that we have not listed?	YES ☐ →	ADD TO TABLE	NO ☐
2B) Are there any other people who may not be members of your family, such as domestic servants, lodgers, or friends who usually live here?	YES ☐ →	ADD TO TABLE	NO ☐
2C) Are there any guests or temporary visitors staying here, or anyone else who stayed here last night, who have not been listed?	YES ☐ →	ADD TO TABLE	NO ☐

CODES FOR Q. 3: RELATIONSHIP TO HEAD OF HOUSEHOLD

01 = HEAD	07 = PARENT-IN-LAW
02 = WIFE OR HUSBAND	08 = BROTHER OR SISTER
03 = SON OR DAUGHTER	09 = OTHER RELATIVE
04 = SON-IN-LAW OR DAUGHTER-IN-LAW	10 = ADOPTED/FOSTER/ STEPCHILD
05 = GRANDCHILD	11 = NOT RELATED
06 = PARENT	98 = DON'T KNOW

HOUSEHOLD SCHEDULE

	IF AGE 0-15 YEARS				IF AGE 5 YEARS OR OLDER		IF AGE 5-24 YEARS		IF AGE 0-4 YEARS
LINE NO.	SURVIVORSHIP AND RESIDENCE OF BIOLOGICAL PARENTS				EVER ATTENDED SCHOOL		CURRENT/RECENT SCHOOL ATTENDANCE		BIRTH REGISTRATION
	**12**	**13**	**14**	**15**	**16**	**17**	**18**	**19**	**20**
	Is (NAME)'s natural mother alive?	Does (NAME)'s natural mother usually live in this household or was she a guest last night?<br>IF YES: What is her name?<br>RECORD MOTHER'S LINE NUMBER.<br>IF NO, RECORD '00'.	Is (NAME)'s natural father alive?	Does (NAME)'s natural father usually live in this household or was he a guest last night?<br>IF YES: What is his name?<br>RECORD FATHER'S LINE NUMBER.<br>IF NO, RECORD '00'.	Has (NAME) ever attended school?	What is the highest level of school (NAME) has attended?<br>What is the highest grade (NAME) completed at that level?<br>SEE CODES BELOW.	Did (NAME) attend school at any time during the [2014-2015] school year?<br>(3)	During [this/that] school year, what level and grade [is/was] (NAME) attending?<br>SEE CODES BELOW.	Does (NAME) have a birth certificate?<br>IF NO, PROBE: Has (NAME)'s birth ever been registered with the civil authority?<br>1 = HAS CERTIFICATE<br>2 = REGISTERED<br>3 = NEITHER<br>8 = DON'T KNOW
	Y N DK		Y N DK		Y N	LEVEL GRADE	Y N	LEVEL GRADE	
01	1 2 8 (GO TO 14)		1 2 8 (GO TO 16)		1 2 (NEXT LINE)		1 2 (NEXT LINE)		
02	1 2 8 (GO TO 14)		1 2 8 (GO TO 16)		1 2 (NEXT LINE)		1 2 (NEXT LINE)		
03	1 2 8 (GO TO 14)		1 2 8 (GO TO 16)		1 2 (NEXT LINE)		1 2 (NEXT LINE)		
04	1 2 8 (GO TO 14)		1 2 8 (GO TO 16)		1 2 (NEXT LINE)		1 2 (NEXT LINE)		
05	1 2 8 (GO TO 14)		1 2 8 (GO TO 16)		1 2 (NEXT LINE)		1 2 (NEXT LINE)		
06	1 2 8 (GO TO 14)		1 2 8 (GO TO 16)		1 2 (NEXT LINE)		1 2 (NEXT LINE)		
07	1 2 8 (GO TO 14)		1 2 8 (GO TO 16)		1 2 (NEXT LINE)		1 2 (NEXT LINE)		
08	1 2 8 (GO TO 14)		1 2 8 (GO TO 16)		1 2 (NEXT LINE)		1 2 (NEXT LINE)		
09	1 2 8 (GO TO 14)		1 2 8 (GO TO 16)		1 2 (NEXT LINE)		1 2 (NEXT LINE)		
10	1 2 8 (GO TO 14)		1 2 8 (GO TO 16)		1 2 (NEXT LINE)		1 2 (NEXT LINE)		

CODES FOR Qs. 24 AND 26: EDUCATION

LEVEL

Level	
00= PRESCHOOL	YEAR 01-02-03
01 = PRIMARY EP1	GRADE 01-05
02 = PRIMARY EP2	GRADE 6-7
3 = SECONDARY ESG1	GRADE 8-10
04 = SECONDARY ESG2	GRADE 11-12
5= ELEMENTARY TECHNICAL SCHOOL	YEAR 01-03
06= BASIC TECHNICAL SCHOOL	YEAR 01-03

LEVEL

Level	
07=INTERMEDIATE TECHNICAL SCHOOL	YEAR 01-03
08 PROFESSIONAL TRAINING BASIC	YEAR01-03
09 = ADVANCED	YEAR 01-07

GRADE

00 = LESS THAN 1 YEAR COMPLETED (ONLY FOR QUESTION 24)

98=DON'T KNOW

HOUSEHOLD CHARACTERISTICS

NO.	QUESTIONS AND FILTERS	CODING CATEGORIES	SKIP
101	What is the main source of drinking water for members of your household?	**PIPED WATER**<br>PIPED INTO DWELLING . . . . . 11<br>PIPED TO YARD/PLOT . . . . . 12<br>PIPED TO NEIGHBOR . . . . . 13<br>PUBLIC TAP/STANDPIPE . . . . . 14<br><br>TUBE WELL OR BOREHOLE . . . . . 21<br>**DUG WELL**<br>PROTECTED WELL . . . . . 31<br>UNPROTECTED WELL . . . . . 32<br>**WATER FROM SPRING**<br>PROTECTED SPRING . . . . . 41<br>UNPROTECTED SPRING . . . . . 42<br><br>RAINWATER . . . . . 51<br>TANKER TRUCK . . . . . 61<br>CART WITH SMALL TANK . . . . . 71<br>SURFACE WATER (RIVER/DAM/ LAKE/POND/STREAM/CANAL/ IRRIGATION CHANNEL) . . . . . 81<br>BOTTLED WATER . . . . . 91<br><br>OTHER ______ 96<br>(SPECIFY)	11–13 → 102<br>14–81 → 103<br><br>96 → 103
102	Where is that water source located?	IN OWN COMPOUND . . . . . 2<br>ELSEWHERE 3	2 → 105
103	How long does it take to go there, get water, and come back?	MINUTES . . . . . [ ][ ][ ]<br><br>DON'T KNOW 998	
104	Do you do anything to the water to make it safer to drink?	YES . . . . . 1<br>NO . . . . . 2<br>DON'T KNOW 8	2, 8 → 109
105	What do you usually do to make the water safer to drink?<br><br>Anything else?<br><br>RECORD ALL MENTIONED.	BOIL . . . . . A<br>ADD BLEACH/CHLORINE . . . . . B<br>STRAIN THROUGH A CLOTH . . . . . C<br>USE WATER FILTER (CERAMIC/ SAND/COMPOSITE/ETC) . . . . . D<br>SOLAR DISINFECTION . . . . . E<br>LET IT STAND AND SETTLE . . . . . F<br><br>OTHER ______ X<br>(SPECIFY)<br>DON'T KNOW . . . . . Z	

No.	Question	Coding categories	Skip
109 (5)	What kind of toilet facility do members of your household usually use?<br><br>IF NOT POSSIBLE TO DETERMINE, ASK PERMISSION TO OBSERVE THE FACILITY.	**FLUSH OR POUR FLUSH TOILET**<br>FLUSH TO PIPED SEWER SYSTEM . . . . 11<br>FLUSH TO SEPTIC TANK . . . . 12<br>FLUSH TO PIT LATRINE . . . . 13<br>FLUSH TO SOMEWHERE ELSE . . . . 14<br>FLUSH, DON'T KNOW WHERE . . . . 15<br>**PIT LATRINE**<br>VENTILATED IMPROVED PIT LATRINE . . . . . 21<br>PIT LATRINE WITH SLAB . . . . 22<br>PIT LATRINE WITHOUT SLAB/OPEN PIT. . . . . 23<br><br>COMPOSTING TOILET . . . . 31<br>BUCKET TOILET . . . . 41<br>HANGING TOILET/HANGING LATRINE . . . . 51<br>NO FACILITY/BUSH/FIELD . . . . 61<br><br>OTHER ______________________ 96<br>(SPECIFY)	61 → 113
110	Do you share this toilet facility with other households?	YES . . . . 1<br>NO 2	2 → 112
111	Including your own household, how many households use this toilet facility?	NO. OF HOUSEHOLDS<br>IF LESS THAN 10 . . . . [0][ ]<br><br>10 OR MORE HOUSEHOLDS . . . . 95<br>DON'T KNOW 98	
112	Where is this toilet facility located?	IN OWN DWELLING . . . . 1<br>IN OWN YARD/PLOT . . . . 2<br>ELSEWHERE 3	

No.	Question		YES	NO
121	Does your household have:			
(7)	Electricity?	ELECTRICITY	1	2
	A radio?	RADIO	1	2
	A television?	TELEVISION	1	2
	A mobile phone?	MOBILE TELEPHONE .	1	2
	A non-mobile phone?	NON-MOBILE TELEPHONE	1	2
	A refrigerator?	REFRIGERATOR	1	2

No.	Question	Coding categories	Skip
110	What type of fuel does your household mainly use for cooking?	ELECTRICITY . . . . 01<br>NATURAL GAS . . . . 03<br>PETROLEUM/PARAFFIN/KEROSENE . . . . 04<br>MINERAL CHARCOAL. . . . . 06<br>CHARCOAL . . . . 07<br>WOOD . . . . 08<br>ANIMAL DUNG . . . . 11<br>NO FOOD COOKED IN HOUSEHOLD . . . . 95<br>OTHER ______________________ 96<br>(SPECIFY)	95 → 113
111	Is the cooking usually done in the house, in a separate building, or outdoors?	IN THE HOUSE . . . . 1<br>IN A SEPARATE BUILDING . . . . 2<br>OUTDOORS . . . . 3<br><br>OTHER ______________________ 6<br>(SPECIFY)	2, 3, 6 → 116
112	Do you have a separate room which is used as a kitchen?	YES . . . . 1<br>NO 2	

NO.		CODING CATEGORIES	SKIP
113<br>(5)	OBSERVE MAIN MATERIAL OF THE FLOOR OF THE DWELLING.<br><br>RECORD OBSERVATION.	**NATURAL FLOOR**<br>EARTH/SAND . . . . 11<br>DUNG . . . . 12<br>**RUDIMENTARY FLOOR**<br>WOOD PLANKS . . . . 21<br>PALM/BAMBOO . . . . 22<br>**FINISHED FLOOR**<br>PARQUET OR POLISHED WOOD . . . . 31<br>VINYL OR ASPHALT STRIPS . . . . 32<br>CERAMIC TILES . . . . 33<br>CEMENT . . . . 34<br>CARPET . . . . 35<br><br>OTHER ______________________ 96<br>(SPECIFY)	
114	OBSERVE MAIN MATERIAL OF THE ROOF OF THE DWELLING.<br><br>RECORD OBSERVATION.	**NATURAL ROOFING**<br>NO ROOF . . . . 11<br>THATCH/PALM LEAF . . . . 12<br>SOD . . . . 13<br>**RUDIMENTARY ROOFING**<br>RUSTIC MAT . . . . 21<br>PALM/BAMBOO . . . . 22<br>WOOD PLANKS . . . . 23<br>CARDBOARD . . . . 24<br>**FINISHED ROOFING**<br>METAL . . . . 31<br>WOOD . . . . 32<br>CALAMINE/CEMENT FIBER . . . . 33<br>CERAMIC TILES . . . . 34<br>CEMENT . . . . 35<br>ROOFING SHINGLES . . . . 36<br><br>OTHER ______________________ 96<br>(SPECIFY)	
115	How many rooms in this household are used for sleeping?	ROOMS . . . . [ ][ ]	
116	Does any member of this household own:<br>a) A watch?<br>b) A mobile phone?<br>c) A bicycle?<br>d) A motorcycle or motor scooter?<br>e) An animal-drawn cart?<br>f) A car or truck?<br>g) A boat with a motor?	YES NO<br>a) WATCH . . . . 1 2<br>b) MOBILE PHONE . . . . 1 2<br>c) BICYCLE . . . . 1 2<br>d) MOTORCYCLE/SCOOTER . . . . . 1 2<br>e) ANIMAL-DRAWN CART . . . . . 1 2<br>f) CAR/TRUCK . . . . 1 2<br>g) BOAT WITH MOTOR 1 2	
117	Does any member of this household own any agricultural land?	YES . . . . 1<br><br>NO 2	 <br>→ 121
118	How many hectares of agricultural land do members of this household own?<br><br>IF 95 OR MORE, CIRCLE '950'.	HECTARES . . . . [ ][ ] . [ ]<br><br>95 OR MORE HECTARES . . . . 950<br>DON'T KNOW 998	
119	Does this household own any livestock, herds, other farm animals, or poultry?	YES . . . . 1<br>NO 2	 → 119
120	How many of the following animals does this household own?<br>IF NONE, RECORD '00'.<br>IF 95 OR MORE, RECORD '95'.<br>IF UNKNOWN, RECORD '98'.<br>Milk cows / bulls?<br>Horses /donkeys?<br>Goats?<br>Ewes / rams?<br>Pigs?<br>Chickens/ ducks?	 <br><br><br>MILK COWS / BULLS<br>HORSES/DONKEYS<br>GOATS<br>EWES/RAMS<br>PIGS<br>CHICKENS/DUCKS	
121	Does any member of this household have a bank account?	YES . . . . 1<br>NO 2	

NO.		NET #1	NET #2	NET #3
127	Does your household have any mosquito nets? How many do you have?			
129 (9)	ASK THE RESPONDENT TO SHOW YOU ALL THE NETS IN THE HOUSEHOLD.<br>IF MORE THAN 3 NETS, USE ADDITIONAL QUESTIONNAIRE(S).	OBSERVED, NO HOLES . . . . . 1<br>OBSERVED, HAS HOLES . . . . 2<br>NOT OBSERVED 3	OBSERVED, NO HOLES . . . . . 1<br>OBSERVED, HAS HOLES . . . . 2<br>NOT OBSERVED 3	OBSERVED, NO HOLES . . . . . 1<br>OBSERVED, HAS HOLES . . . . 2<br>NOT OBSERVED 3
130 (9)	How many months ago did your household get the mosquito net?<br>IF LESS THAN ONE MONTH AGO, RECORD '00'.	MONTHS AGO . . . . . [ ][ ]<br>MORE THAN 36 MONTHS AGO . . . . . 95<br>NOT SURE 98	MONTHS AGO . . . . . [ ][ ]<br>MORE THAN 36 MONTHS AGO . . . . . 95<br>NOT SURE 98	MONTHS AGO . . . . . [ ][ ]<br>MORE THAN 36 MONTHS AGO . . . . . 95<br>NOT SURE 98
131 (9)	OBSERVE OR ASK BRAND/TYPE OF MOSQUITO NET.<br>IF BRAND IS UNKNOWN AND YOU CANNOT OBSERVE THE NET, SHOW PICTURES OF TYPICAL NET TYPES/BRANDS TO RESPONDENT.	**LONG-LASTING INSECTICIDE-TREATED NET (LLIN)**<br>BRAND A . . . . 11<br>BRAND B . . . . 12<br>OTHER/DON'T KNOW BRAND . . . . 16<br>(SKIP TO 134)<br>OTHER TYPE . . . . 96<br>DON'T KNOW TYPE . . 98	**LONG-LASTING INSECTICIDE-TREATED NET (LLIN)**<br>BRAND A . . . . 11<br>BRAND B . . . . 12<br>OTHER/DON'T KNOW BRAND . . . . 16<br>(SKIP TO 134)<br>OTHER TYPE . . . . 96<br>DON'T KNOW TYPE . . 98	**LONG-LASTING INSECTICIDE-TREATED NET (LLIN)**<br>BRAND A . . . . 11<br>BRAND B . . . . 12<br>OTHER/DON'T KNOW BRAND . . . . 16<br>(SKIP TO 134)<br>OTHER TYPE . . . . 96<br>DON'T KNOW TYPE . . 98
136 (9)	Did anyone sleep under this mosquito net last night?	YES . . . . 1<br>NO . . . . 2<br>(SKIP TO 138)<br>NOT SURE 8	YES . . . . 1<br>NO . . . . 2<br>(SKIP TO 138)<br>NOT SURE 8	YES . . . . 1<br>NO . . . . 2<br>(SKIP TO 138)<br>NOT SURE 8
137 (9)	Who slept under this mosquito net last night?	Participant 1<br>Spouse 2<br>Other adult 3<br>Child aged 5-14 years 4<br>Child under 5 years 5	Participant 1<br>Spouse 2<br>Other adult 3<br>Child aged 5-14 years 4<br>Child under 5 years 5	Participant 1<br>Spouse 2<br>Other adult 3<br>Child aged 5-14 years 4<br>Child under 5 years 5
		Participant 1<br>Spouse 2<br>Other adult 3<br>Child aged 5-14 years 4<br>Child under 5 years 5	Participant 1<br>Spouse 2<br>Other adult 3<br>Child aged 5-14 years 4<br>Child under 5 years 5	Participant 1<br>Spouse 2<br>Other adult 3<br>Child aged 5-14 years 4<br>Child under 5 years 5
		Participant 1<br>Spouse 2<br>Other adult 3<br>Child aged 5-14 years 4<br>Child under 5 years 5	Participant 1<br>Spouse 2<br>Other adult 3<br>Child aged 5-14 years 4<br>Child under 5 years 5	Participant 1<br>Spouse 2<br>Other adult 3<br>Child aged 5-14 years 4<br>Child under 5 years 5
		GO BACK TO 121 FOR NEXT NET; OR, IF NO MORE NETS, GO TO 128.	GO BACK TO 121 FOR NEXT NET; OR, IF NO MORE NETS, GO TO 128.	GO TO 121 IN FIRST COLUMN OF A NEW QUESTIONNAIRE; OR, IF NO MORE NETS, GO TO 128.

ADDITIONAL HOUSEHOLD CHARACTERISTICS

NO.	QUESTIONS AND FILTERS	CODING CATEGORIES	SKIP
128	We would like to learn about the places that households use to wash their hands. Can you please show me where members of your household most often wash their hands?	OBSERVED, FIXED PLACE . . . . 1<br>OBSERVED, MOBILE . . . . 2<br>NOT OBSERVED,<br>NOT IN DWELLING/YARD/PLOT . . . . 3<br>NOT OBSERVED, NO PERMISSION TO SEE . . . . . 4<br>NOT OBSERVED, OTHER REASON 5	3, 4, 5 → 142
129	OBSERVE PRESENCE OF WATER AT THE PLACE FOR HANDWASHING.<br><br>RECORD OBSERVATION.	WATER IS AVAILABLE . . . . 1<br>WATER IS NOT AVAILABLE 2	
130	OBSERVE PRESENCE OF SOAP, DETERGENT, OR OTHER CLEANSING AGENT AT THE PLACE FOR HANDWASHING.<br><br>RECORD OBSERVATION.	SOAP OR DETERGENT<br>(BAR, LIQUID, POWDER, PASTE) . . . . A<br>ASH, MUD, SAND . . . . B<br><br>NONE . . . . Y	
**INJURIES AND TRAUMA.**			
131	How many members of the household have been injured in a car accident in the last 6 months (whether as occupants or as pedestrians)?	NUMBER<br>NUMBER OF ADULTS<br>NUMBER OF CHILDREN	
132	Please indicate how seriously each of those involved in such an accident was intjured.	INJURED<br>HOSPITALIZED<br>PERMANENTLY DISABLED<br>DIED AT THE SCENE<br>DIED AT THE HOSPITAL<br>DIED IMMEDIATELY	
133	How many members of the household have been injured in some other fashion in the last 6 months?	NUMBER<br>NUMBER OF ADULTS<br>NUMBER OF CHILDREN	
134	What was the cause or manner of these injuries?	FALL<br>BLOW / ASSAULT<br>CUT / STABBING<br>HANGING/STRANGLING<br>SHOT WITH FIREARM<br>POISONING / VENOM<br>SEXUAL ASSAULT<br>BITE<br>BURN<br>OTHER	

MANICA AND SOFALA COMMUNITY SURVEY (InCoMaS) INDIVIDUAL QUESTIONNAIRE

SECTION 0 IDENTIFICATION

QUESTIONS	CODING CATEGORIES
GPS COORDINATES	LATITUDE………………………............................ □□□□□□<br>LONGITUDE……………………………………………. □□□□□□
HOUSEHOLD STUDY CODE	URBAN…………………………………………………... 1<br>RURAL…………………………………………………... 2
HOUSEHOLD SELECTED FOR THE FOLLOWING MODULES	**Maternal and child health**<br>Children aged 0-4…………………………………….. □<br>Children aged 5-14…………………………………… □<br>Mother of children under 5…………………………..... □<br>Other women of childbearing age, 15-49………….. □<br>**Non-transmissible diseases and cardiovascular risk**<br>Men aged 15 years and older……………………….. □<br>Women aged 50 years and older………………….…... □

SECTION 1 CHARACTERISTICS OF THE RESPONDENT

NO.	QUESTIONS	CODING CATEGORIES	SKIP
201	Note the time	HOURS…………………………….... □□<br>MINUTES……………………………<br>. □□	
202	In which month and year were you born?	MONTH…………………………….... □□<br>MONTH NOT KNOWN…………… 98<br>YEAR……………………………….. □□<br>YEAR NOT KNOWN……………… 9998	
203	What is your age in years?<br><br>COMPARE 201 AND 202 AND CORRECT IF INCONSISTENCIES ARE FOUND	AGE IN YEARS…………………… □□	
204	Did you ever attend school?	YES……………………................ 1<br>NO…………………………………… 2	
205	What was the highest level of schooling you attended?	LITERACY……………… 00<br>LOWER PRIMARY……………… 01<br>UPPER PRIMARY…………… 02<br>LOWER SECONDARY…………… 03<br>UPPER SECONDARY…………… 04<br>ELEMENTARY TECHNICIAN… 05<br>BASIC TECHNICIAN……………… 06<br>INTERMEDIATE TECHNICIAN.… 07<br>TEACHING CERTIFICATE…….. 08<br>HIGHER………………………….. 09	
206	What was the highest grade / year you completed at this level?<br><br>IF SUBJECT DID NOT COMPLETE A FULL GRADE OR YEAR, WRITE '00'.	CLASS / YEAR…………………….. □□	
207	What is your religion?	CATHOLIC 01<br>MUSLIM 02<br>ZIONE/SIÃO 03<br>EVANGELICAL/PETENCOSTAL 04<br>ANGLICAN 05<br>JOHANMARANGA 06<br>NO RELIGION 07<br>OTHER______________________ 96<br>(SPECIFY)	
208	In which language did you learn to speak?	PORTUGUeSE 01<br>BITONGA 02<br>CINYUNGWE 03	

NO.	QUESTIONS	CODING CATEGORIES		SKIP
		CISENA<br>CINDAU<br>CITEWE<br>SHONA<br>ELOMWE<br>ECHUWABO<br>XICHANGANA<br>EMAKHUWA<br>OTHER______________________.<br>(SPECIFY)	04<br>05<br>06<br>07<br>08<br>09<br>10<br>11<br>96	
209	In the last 12 months, how often did you spend one or more nights away from your home?	NUMBER OF TIMES<br>NEVER	□□<br>00	
210	In the last 12 months, did you ever spend a month or more out of the house?	YES……………………….............<br>NO……………………………………	1<br>2	
211	WEIGHT IN KILOGRAMS	KG……………………………………<br>ABSENT…………………………….<br>REFUSED………………………….<br>OTHER………………………………	□□□<br>888<br>999<br>666	
212	HEIGHT IN CENTIMETERS	CM…………………………………...<br>ABSENT…………………………….<br>REFUSED………………………….<br>OTHER………………………………	□□□<br>888<br>999<br>666	
213	BLOOD PRESSURE	Mm Hg………………………………	□□□	

Women aged 15-49: go to SECTION 2.
Parent / legal guardian of a child under 5: go to SECTION 4
Women aged 50+: go to SECTION 5
Men aged 15+: go to SECTION 5

SECTION 2 REPRODUCTIVE HISTORY (for women)

NO.	QUESTIONS	CODING CATEGORIES		SKIP
	**I'd now like to ask you some questions about your live-born sons and daughters.**			
220	Do you have any living children?	YES……………………….............<br>NO……………………………………	1<br>2	
221	Do you have any children living with you?	YES……………………….............<br>NO……………………………………	1<br>2	
222	How many sons do you have who live with you?<br><br>How many daughters do you have who live with you?<br><br>IF NONE, WRITE '00'.	SONS LIVING HERE……………....<br><br>DAUGHTERS LIVING HERE…….	□□<br><br>□□	
223	Do you have any children who do not live in your home?	YES……………………….............<br>NO……………………………………	1<br>2	
224	How many sons do you have who do not live with you?<br><br>How many daughters do you have who do not live with you?<br><br>IF NONE, WRITE '00'.	SONS LIVING ELSEWHERE ……<br><br>DAUGHTERS LIVING ELSEWHERE…………..............	□□<br><br>□□	
225	Did you ever have a live-born son or daughter who later died?	YES……………………….............<br>NO……………………………………	1<br>2	If 1, 226<br>If 2, 225A
225a	Did you ever have a baby who cried or showed signs of life, but who only lived a few hours or days?	YES……………………….............<br>NO……………………………………	1<br>2	Go to 227
226	How many sons do you have who are deceased?<br><br>How many daughters do you have who are deceased?<br><br>IF NONE WRITE '00'.	DECEASED SONS………………..<br><br>DECEASED DAUGHTERS…………...	□□<br><br>□□	
227	SUM RESPONSES TO 222, 224, AND 226, AND NOTE THE TOTAL.<br><br>IF NONE WRITE '00'.	TOTAL LIVE-BORN CHILDREN…	□□	
228	**CONSULT 227:**<br>Just to make sure I understood correctly:<br>You had a total of ____live-born children in your life?	YES……………………….............<br>NO……………………………………	1<br>2	If 2, verify and correct from 220

NO.	QUESTIONS	CODING CATEGORIES		SKIP
				to 226 if necessary
229	**CONSULT 227:**	ONE OR MORE LIVE-BORN……..<br>NO LIVE-BORN CHILDREN……….	1<br>2	If 2, go to 244.
	**INTERVIEWER: ASK FOR EACH CHILD'S NAME AND USE WHEN ASKING THE FOLLOWING QUESTIONS**			
230	What is your first child's name?<br><br>(After 241) What is your next child's name?	________________________		
231	What is (NAME)'s sex?	Male…………………………………<br>Female………………………………	1<br>2	
232	Is (NAME) a twin?	Not a twin/single birth<br>Twin/multiple birth	1<br>2	
233	In which month and year was (NAME) born?<br><br>*CLARIFY: When is his/her birthday?*	MONTH_________________<br>YEAR__________________		
234	Is (NAME) alive?	YES……………………..............<br>NO……………………………………	1<br>2	If 2, go to 238
235	How old was (NAME) on his / her last birthday?<br><br>NOTE THE AGE IN YEARS	AGE IN YEARS:_____________		
236	Does (NAME) live with you?	YES……………………..............<br>NO……………………………………	1<br>2	
237	LIST CHILD'S BIRTH ORDER	BIRTH ORDER_________		Go to 239
238	How old was (NAME) when he / she died?<br><br>IF LESS THAN 2 YEARs, ASK<br>How many months old was (NAME)?<br><br>*NOTE: Choose unit; days if less than 1 month, months if less than 2 years and years if more than 2 years old*	Child's age: ………………………<br><br>UNITS:<br>Days…………………………………<br>Months………………………………<br>Years………………………………..	□□<br><br><br>1<br>2<br>3	
239	Was there another birth in between (NAME) and the last child we discussed, including children who died soon after birth?	YES……………………..............<br>NO……………………………………	1<br>2	If 1, add births.<br>If 2, go to next birth
240	Did you have another child after the birth of (LAST CHILD´S NAME)?	YES……………………..............<br>NO……………………………………	1<br>2	If 1, go back and complete the birth history
241	COMPARE 227 WITH THE NUMBER OF CHILDEN MENTIONED AND CHECK:<br><br>(VERIFY AND CORRECT INCONSISTENCIES)	THE NUMBERS ARE THE SAME…<br><br>THE NUMBERS ARE DIFFERENT..	1<br><br>2	If 1, go to 242.<br>If 2, go to 230
242	NOTE THE NUMBER OF BIRTHS IN 2011 OR LATER	NUMBER OF BIRTHS......................	□□	If 0, go to 244
243	FOR EACH BIRTH SINCE JANUARY 2011, NOTE THE MONTH OF BIRTH AND ASK FOR DURATION OF THE PREGNANCY	MONTH OF BIRTH_________________<br><br>DURATION OF THE PREGNANCY______		
244	Are you currently pregnant?	YES……………………..............<br>NO……………………………………<br>NOT SURE..……………………….	1<br>2<br>3	If 2 or 3, go to 246.
245	How many months pregnant are you?<br>NOTE NUMBER OF MONTHS	MONTHS..…………………………..	□□	
246	Did you ever have a pregnancy that was lost (through miscarriage or stillbirth)?	YES……………………..............<br>NO……………………………………	1<br>2	If 2, go to 248
247	In which month and year did that pregnancy end?	MONTH..…………………………….<br>YEAR..……………………………….	□□<br>□□□<br>□	
248	**CONSULT 233**<br>LAST PREGNANCY ENDED	IN JAN.2011 OR LATER………….<br>BEFORE JAN 2011..………………	1<br>2	If 1, go to 249a.<br>If 2, go to 251
249a	How many months pregnant were you when the pregnancy terminated?<br>NOTE THE NUMBER OF MONTHS COMPLETED.	MONTH..…………………………….	□□	
249b	Did you have another pregnancy that ended in loss (miscarriage or stillbirth) since JANUARY 2011?	YES……………………..............<br>NO……………………………………	1<br>2	If 2, go to 251

NO.	QUESTIONS	CODING CATEGORIES	SKIP
250	ASK FOR THE DATE AND DURATION OF EACH PREGNANCY THAT ENDED IN MISCARRIAGE OR STILLBIRTH SINCE JANUARY 2011	DATE_________________<br><br>DURATION OF THE PREGNANCY______	
251	Did you ever have a pregnancy that ended in loss (miscarriage or stillbirth) before 2011?	YES……………………….............. 1<br>NO…………………………………… 2	
252	In which month and year did that pregnancy end?	MONTH..……………………………. □□<br>YEAR..………………………………. □□□□	

SECTION 3 PREGNANCY, POST-PARTUM CARE AND FAMILY PLANNING

NO.	QUESTIONS	CODING CATEGORIES	SKIP
253	*NOTE THE NAME, BIRTH ORDER, AND VITAL STATUS OF EACH CHILD BORN SINCE JANUARY 2011. ASK THE QUESTIONS ABOUT EACH LIVE-BORN CHILD, STARTING WITH THE MOST RECENT.*<br><br>I'd now like to ask you some questions about your children's health in the last five years. We'll discuss the children one at a time.		
254	BIRTH ORDER	BIRTH ORDER REPORTED IN BIRTH HISTORY SECTION …… □□	
255	CHILD'S VITAL STATUS (SEE 234)	___________ ___________<br>ALIVE DECEASED	
256	When you became pregnant with (NAME) did you want to become pregnant?	YES……………………….............. 1<br>NO…………………………………… 2	If 1, go to 259
257	Did you want a child later, or not at all?	WANTED A CHILD LATER……… 1<br>DIDN'T WANT ANY / ANY MORE CHILDREN………………………… 2	If 2, go to 259
258	How long did you want to wait?	Amount of time (in months/ years). □□<br>Not sure………………………… 998<br><br>UNITS:<br>Months....................................... 1<br>Years... 2	
259	Did you have any antenatal care during this pregnancy?	YES……………………….............. 1<br>NO…………………………………… 2	If 2, go to 415
260	Who examined you?	**MEDICAL PROFESSIONAL**<br>DOCTOR.................................. 1<br>SURGICAL TECH..................... 2<br>MEDICAL TECH....................... 3<br>NURSE .... 4<br>MIDWIFE .... 5<br><br>**OTHER**<br>TRADITIONAL BIRTH ATTENDANT............................ 6<br><br>OTHER___________________ 10<br>(SPECIFY)	
261a	Where did you do antenatal care consults?<br><br>ASK TO DETERMINE THE CATEGORY	**PRIVATE HOUSE**<br>SUBJECT'S HOME .... 11<br>SOMEONE ELSE'S HOME........ 12<br><br>**PUBLIC SECTOR** 21<br>CENTRAL HOSPITAL ....<br>PROVINCIAL/GENERAL 22<br>HOSPITAL.................................... 23<br>RURAL HOSPITAL................... 24<br>CLINIC / HEALTH CENTER…... 25<br>MOBILE HEALTH BRIGADE….. 26<br>OTHER _________________....<br>(SPECIFY)<br><br>**PRIVATE SECTOR** 31<br>CLINIC....................................... 32<br>MEDICAL CONSULT .... 33<br>NURSE .... 34<br>PHARMACY..............................	

NO.	QUESTIONS	CODING CATEGORIES		SKIP
		OTHER___________________..... (SPECIFY)	98	
261b	RECORD NAME OF THE PLACE WHERE SUBJECT DID ANTENATAL CARE	___________________________ Name of clinic		
262	How many months pregnant were you when you had your first antenatal care visit?	MONTH... NOT SURE...................................	□□ 98	
263	How many antenatal care visits did you have during the pregnancy?	NUMBER OF CONSULTS……….. NOT SURE....................................	□□ 98	
264	During your antenatal care visits, did the following occur:			
264a	BLOOD PRESSURE MEASUREMENT?	YES……………………................ NO……………………………………	1 2	
264b	URINE TEST?	YES……………………................ NO……………………………………	1 2	
264c	BLOOD TEST?	YES……………………................ NO……………………………………	1 2	
265	Who attended (NAME)'s birth?	**MEDICAL PROFESSIONAL** DOCTOR................................ SURGICAL TECH.................... MEDICAL TECH...................... NURSE .... MIDWIFE .... <br>**OTHER PERSON** TRADITIONAL BIRTH ATTENDANT.............................. FRIENDS / FAMILY MEMBERS.. <br>OTHER __________________...... (SPECIFY) <br>NO ONE..	 1 2 3 4 5 <br><br><br>6 7 <br>10 <br>98	
266a	Where was (NAME) born? <br>ASK TO DETERMINE THE CATEGORY <br>NOTE THE NAME OF THE PLACE <br>____________________________	**PRIVATE HOUSE** SUBJECT'S HOME…………….. SOMEONE ELSE'S HOME…… <br>**PUBLIC SECTOR** CENTRAL HOSPITAL ………… PROVINCIAL/GENERAL……… HOSPITAL………………….….... RURAL HOSPITAL.................... CLINIC / HEALTH CENTER OTHER _______________...... (SPECIFY) <br>**PRIVATE SECTOR** CLINIC..................................... MEDICAL CONSULT .... NURSE .... PHARMACY .... <br>OTHER __________________...... (SPECIFY)	 11 12 <br><br>21 <br>22 23 24 26 <br><br>31 32 33 34 <br>98	If 11, go to 270
267	Was (NAME) born via Cesarian section, that is to say, were you operated on to extract the baby?	YES……………………................ NO……………………………………	1 2	
268	After (NAME) was born, did anyone observe you to monitor your health status, while you were still in the health facility?	YES................................. NO................................	1 2	If 1, go to 439
269	Who observed you?	**MEDICAL PROFESSIONAL** DOCTOR……………………….. SURGICAL TECH.................... MEDICAL TECH...................... NURSE .... MIDWIFE .... <br>**OTHER PERSON** TRADITIONAL BIRTH ATTENDANT……………………….	 1 2 3 4 5 <br><br><br>6	

NO.	QUESTIONS	CODING CATEGORIES		SKIP
		OTHER ________________...... (SPECIFY)	98	
270	Was (NAME) ever breastfed?	YES……………………............<br>NO……………………………………	1<br>2	If 2, go to 274
271	Is (NAME) currently breastfed?	YES……………………............<br>NO……………………………………	1<br>2	If 1, go to 274
272	How old was (NAME) when he / she stopped breastfeeding?	MONTHS……………………………<br>YEARS………………………………<br>NOT SURE..................................	□□<br>□□<br>98	
273	How old was (NAME) when he/she began drinking other liquids besides breast milk?	LESS THAN 1 MONTH……………<br>MONTHS……………………………<br>STILL DOES NOT DRINK OTHER LIQUIDS……………………………<br>NOT SURE..................................	0<br>□□<br>88<br>98	
274	How old was (NAME) when he/she began eating solids?	LESS THAN 1 MONTH……………<br>MONTHS……………………………<br>STILL DOES NOT DRINK OTHER LIQUIDS……………………………<br>NOT SURE..................................	0<br>□□<br>88<br>98	
275	Ideally, how long should babies be exclusively breastfed?	LESS THAN 1 MONTH……………<br>MONTHS……………………………<br>NOT SURE..................................	0<br>□□<br>98	

NO.	QUESTIONS	CODING CATEGORIES		SKIP
	Now I'd like to discuss family planning methods - the ways or methods that couples use to delay or prevent pregnancy. I'd like to know which methods you use, as well as the once you know or have heard of.			
276	Do you currently use a method to delay or prevent pregnancy?	YES……………………............<br>NO……………………………………	1<br>2	
277	Have you ever used or tried to use a method to delay or prevent pregnancy?	YES……………………............<br>NO……………………………………	1<br>2	
278	Which method do you currently use (or which was the most recent method you used)?	FEMALE STERILIZATION……….<br>MALE STERILIZATION…………..<br>IUD………………………………….<br>INJECTABLES……………………<br>IMPLANTS………………………..<br>PILL………………………………..<br>CONDOM…………………………<br>FEMALE CONDOM………………<br>LACTATIONAL AMENORRHEA METHOD …………………………..<br>RHYTHM METHOD……………...<br>WITHDRAWAL……………………<br>OTHER MODERN METHOD……<br>OTHER TRADITIONAL METHOD.	A<br>B<br>C<br>D<br>E<br>F<br>G<br>H<br>I<br>J<br>K<br>X<br>Y	If A,B,C,D,E, go to 280<br>If F, go to 279<br>If G, H, I, J, K, X, Y, go to 282
279	What kind of pill did you use?<br>IF SUBJECT DOESN'T KNOW THE BRAND, ASK TO SEE A PACKAGE	MICROGENON……………………<br>MICROLUT…………………………<br>OTHER ________________...... (SPECIFY)<br>NOT SURE…………………………	1<br>2<br>6<br>98	
280	Where did you acquire the (CURRENT / MOST RECENT METHOD) when you first started using it?	**PUBLIC SECTOR**<br>CENTRAL HOSPITAL ....<br>PROVINCIAL/GENERAL HOSPITAL…………………………<br>RURAL HOSPITAL……………..<br>CLINIC / HEALTH CENTER…..<br>MOBILE HEALTH BRIGADE….<br>PHARMACY……………………<br>OTHER ______________...... (SPECIFY)<br>**PRIVATE SECTOR**<br>CLINIC....................................	 11<br>12<br>13<br>14<br>16<br>17<br>20<br><br>22	

NO.	QUESTIONS	CODING CATEGORIES		SKIP
		DOCTOR................................<br>NURSE ………………………....<br>PHARMACY …………………..<br>SHOP.....................................<br>PETROL STATION……………<br>BAR/DISCO.............................<br>VENDOR'S TENT.....................<br>OTHER ________________......<br>(SPECIFY)<br><br>**OTHER SOURCES**<br>SCHOOL<br>MARKET<br>CHURCH<br>FRIENDS/FAMILY<br>TRADITIONAL HEALER<br>ADOLESCENT HEALTH SERVICES<br><br>OTHER ________________......<br>(SPECIFY)	23<br>24<br>25<br>26<br>27<br>28<br>29<br>30<br><br><br><br>32<br>33<br>34<br>35<br>37<br>38<br><br>40	
281	IF CURRENTLY USING:<br>How long have you used (CURRENT METHOD) continuously?<br><br>IF NOT CURRENTLY USING:<br>For how long did you use (MOST RECENT METHOD) continuously?	YEARS...<br><br>MONTHS ……………………………...	□□<br><br>□□	

NO.	QUESTIONS	CODING CATEGORIES	Y	N	SKIP
282	Do you know of / have you heard of any of these contraceptive methods?	FEMALE STERILIZATION……….	1	2	
		MALE STERILIZATION…………	1	2	
		IUD…………………………………	1	2	
		INJECTABLES ……………………	1	2	
		IMPLANTS…………………………	1	2	
		PILL…………………………………	1	2	
		CONDOM…………………………	1	2	
		FEMALE CONDOM ……………..	1	2	
		LACTATIONAL AMENORRHEA METHOD……………………………	1	2	
		RHYTHM METHOD………………..	1	2	
		WITHDRAWAL……………………..	1	2	
		OTHER MODERN METHOD……..<br>________________<br>(SPECIFY)	1	2	
		OTHER TRADITIONAL METHOD.<br>________________<br>(SPECIFY)	1	2	

SECTION 4 CHILD HEALTH

NO.	QUESTIONS	CODING CATEGORIES		SKIP
285	INTERVIEWER: ASK FOR EACH CHILD'S NAME BEFORE YOU BEGIN READING THE QUESTIONS, JUST TO GUIDE THE RESPONDENT, ASK FOR THE CHILD'S HEALTH CARD. REPEAT QUESTIONS IN THIS SECTION FOR EACH CHILD			
286	What is the child's birth order number?	BIRTH ORDER……………………..	□□	
287	What is the child's sex?	FEMALE……………………………<br>MALE……………………………….	1<br>2	
288	Weight in kilograms	KG…………………………………..<br>ABSENT……………………………<br>REFUSED………………………….<br>OTHER ________________.........<br>(SPECIFY)	□□□<br>996<br>997<br>998	

NO.	QUESTIONS	CODING CATEGORIES		SKIP
289	Height in centimeters	CM. …………………………………<br>AUSENTE. …………………………<br>RECUSOU…………………………<br>OTHER ________________..........<br>(SPECIFY)	□□□<br>996<br>997<br>998	
290	Was height measured standing or reclined?	RECLINED…………………………<br>STANDING…………………………<br>NOT MEASURED …………………	1<br>2<br>98	
291	ASK THE CHILD'S GUARDIAN:<br>What is (name)'s birth date?	DAY…………………………………<br>MONTH……………………………<br>YEAR……………………………….	□□<br>□□<br>□□□□	
292	Consult 291:<br>Was the child born in January 2011 or later??	YES……………………….............<br>NO……………………………………	1<br>2	Go to 286 for the next child, or if there are no more, go to 293
293	NOTE THE NUMBER AND VITAL STATUS OF EACH CHILD BORN IN 2011 OR LATER. ASK THE QUESTIONS FOR ALL LIVE-BORN CHILDREN, STARTING WITH THE YOUNGEST.			
294	BIRTH ORDER	NO OF BIRTH ORDER……………	□□	
295	CHILD'S VITAL STATUS	ALIVE……………………………..…<br>DECEASED…………………………	1<br>2	If 2, go to 294 for next child or, if there are no more births, go to the next module
296	Does (NAME) have a health card?<br>IF YES: Can I see it?	YES, SAW CARD…………………......<br>YES, DID NOT SEE CARD………..<br>DOES NOT HAVE CARD…………	1<br>2<br>3	If 1, go to 298.
297	Did (NAME) ever have a health card?	YES……………………….............<br>NO……………………………………	1<br>2	Go to 300
298	(1) FOR EACH VACCINE, COPY THE DATES FROM THE HEALTH CARD<br>(2) WRITE '44' IN THE "DAY" COLUMN IF THE CARD SHOWS THE CHILD RECEIVED THE VACCINE BUT DOESN'T SHOW DATE			

	DAY	MONTH	YEAR
**BCG**			
**POLIO AT BIRTH**			
**DPT/HEPATITIS B1**			
**POLIO 1**			
**PCV-10 1**			
**ROTAVIRUS 1**			
**DPT / HEPATITIS B 2**			
**PCV-10 2**			
**POLIO 2**			
**ROTAVIRUS 2**			
**DPT / HEPATITIS B 3**			
**POLIO 3**			
**PCV-10 3**			
**MEASLES 1**			
**MEASLES 2**			
**VITAMINA A (LAST DOSE)**			

NO.	QUESTIONS	CODING CATEGORIES		SKIP
299	Did (NAME) receive any vaccines that do not appear in their health card, including vaccines received in mass immunization campaigns?<br><br>NOTE YES ONLY IF THE RESPONDENT RESPONDED BCG, POLIO, DPT 1-3, MEASLES OR VITAMIN A	YES………………………………….<br>IF YES, ASK WHICH VACCINES AND NOTE '66' IN THE DAYS COLUMN IN 298.<br>NO……………………………………<br>NOT SURE…………………………	1<br><br><br><br><br>2<br>8	Go to 302
300	Did (NAME) receive any vaccines to prevent illness, including vaccines received in mass immunization campaigns?	YES……………………….............<br>NO……………………………………<br>NOT SURE…………………………	1<br>2<br>8	If 2 or 8, go to 302

NO.	QUESTIONS	CODING CATEGORIES	SKIP
301	Please tell me if (NAME) received any of the following:		
301A	Has (NAME) ever received a BCG vaccination against tuberculosis, that is, an injection in the arm or shoulder that usually causes a scar?	YES……………………................ 1<br>NO…………………………………… 2<br>NOT SURE………………………… 8	
301B	Has (NAME) ever received oral polio vaccine, that is, about two drops in the mouth to prevent polio?	YES……………………................ 1<br>NO…………………………………… 2<br>NOT SURE………………………… 8	If 2 or 8, go to 301E
301C	Did (NAME) receive the first oral polio vaccine in the first two weeks after birth or later?	FIRST WEEK……………………… 1<br>LATER…………………………… 2	
301D	How many times did (NAME) receive the oral polio vaccine?	NUMBER OF TIMES……………… □	
301E	Has (NAME) ever received a pentavalent vaccination, that is, an injection given in the thigh sometimes at the same time as polio drops?	YES……………………................ 1<br>NO…………………………………… 2<br>NOT SURE………………………… 8	If 2 or 8, go to 301g
301F	How many times did (NAME) receive the pentavalent vaccine?	NUMBER OF TIMES……………… □	
301G	Has (NAME) ever received a measles vaccination, that is, an injection in the arm to prevent measles?	YES……………………................ 1<br>NO…………………………………… 2<br>NOT SURE………………………… 8	
301H	PCV-10, that is, an injection to prevent pneumonia?	YES……………………................ 1<br>NO…………………………………… 2<br>NOT SURE………………………… 8	
302	Did (NAME) receive a dose of vitamin A in the last 6 months?<br><br>SHOW SOME AMPULES/CAPSULES	YES……………………................ 1<br>NO…………………………………… 2<br>NOT SURE………………………… 8	
303	In the last 7 days, did (NAME) take iron salts like these?<br><br>SHOW SOME AMPULES/CAPSULES	YES……………………................ 1<br>NO…………………………………… 2<br>NOT SURE………………………… 8	
304	Did (NAME) take any antiparasitic drugs in the last 6 months?	YES……………………................ 1<br>NO…………………………………… 2<br>NOT SURE………………………… 8	
305	To which hospital or clinic do you take (NAME) for child-at-risk services?	Child doesn't go to child-at-risk services……………………….…..... 0<br>Name of clinic…………………….. □	
306	Has (NAME) had diarrhea in the last two weeks?	YES……………………................ 1<br>NO…………………………………… 2<br>NOT SURE………………………… 8	If 2, go to 320
307	Did he / she have blood in their stool?	YES……………………................ 1<br>NO…………………………………… 2<br>NOT SURE………………………… 8	
308	Did you get medical advice or treatment for the diarrhea?	YES……………………................ 1<br>NO…………………………………… 2	If 2, go to 320
309	Where did you seek medical advice or treatment?<br><br>Anywhere else?<br><br>NOTE ALL RESPONSES<br><br>____________________<br>(NAME OF PLACE)	**PUBLIC SECTOR**<br>HEALTH FACILITY.................... A<br>MOBILE HEALTH BRIGADE…. B<br>OTHER PUBLIC________........ C<br>(SPECIFY)<br><br>**PRIVATE SECTOR**<br>CLINIC....................................... D<br>PHARMACY.............................. E<br>DOCTOR..................... ……….. F<br>OTHER ________________...... G<br>(SPECIFY)<br><br>**OTHER LOCATION**<br>MARKET....................................... H<br>PRACTICIONER OF TRADITIONAL MEDICINE……….. I<br>NEIGHBORHOOD HEALTH PERSONELL………………………. J<br>OTHER ________________......... X<br>(SPECIFY)	
310	Was (NAME) given any of the following at any time since (NAME) started having the diarrhea:		

NO.	QUESTIONS	CODING CATEGORIES	SKIP
	a) A fluid made from a special packet called ORS?<br>b) A homemade fluid made of water, salt and sugar?<br>c) Rice water?	YES NO NOT SURE<br>a) ORS………… 1 2 8<br>b) WATER, SALT SUGAR… 1 2 8<br>c) RICE WATER. 1 2 8	
311	Was anything else given to treat the diarrhea?	YES……………………............ 1<br>NO…………………………………… 2<br>NOT SURE………………………… 8	If 2 or 8, go to 320
312	What was given to treat the diarrhea?<br><br>Anything else?<br><br>NOTE ALL TREATMENTS	TABLETS/ SYRUPS.......................... A<br>INJECTIONS.................................... B<br>IV FLUIDS... C<br>HOUSE REMEDY / HERBAL MEDICINE .... D<br>OTHER ________________......... X<br>(SPECIFY)	
320	Has (NAME) been ill with a fever at any time in the last 2 weeks?	YES……………………............ 1<br>NO…………………………………… 2<br>NOT SURE………………………… 8	If 2 or 8, please go to 322B
321	At any time during the illness, did (NAME) have blood taken from (NAME)'s finger or heel for testing?	YES……………………............ 1<br>NO…………………………………… 2<br>NOT SURE………………………… 8	
322A	When (NAME) had a fever, was it accompanied by cough?	YES……………………............ 1<br>NO…………………………………… 2<br>NOT SURE………………………… 8	If 1, go to 323.<br>If 2 or 8, go to 325
322B	Has (NAME) had an illness with a cough at any time in the last 2 weeks?	YES……………………............ 1<br>NO…………………………………… 2<br>NOT SURE………………………… 8	If 2 or 8, go to 325
323	Has (NAME) had fast, short, rapid breaths or difficulty breathing at any time in the last 2 weeks?	YES……………………............ 1<br>NO…………………………………… 2<br>NOT SURE………………………… 8	If 2 or 8, go to 325
324	Was the fast or difficult breathing due to a problem in the chest or to a blocked or runny nose?	CHEST ONLY............................... 1<br>NOSE ONLY................................. 2<br>BOTH.. 3<br>OTHER ________________......... 6<br>(SPECIFY) NOT SURE.. 8	
325	Now I would like know how much (NAME) was given to drink during the illness, including breastmilk. Was (NAME) given less than usual to drink, about the same amount, or more than usual to drink?<br><br>IF LESS, ASK: A little less or a lot less?	MUCH LESS................................. 1<br>A LITTLE LESS............................ 2<br>SAME AMOUNT........................... 3<br>MORE... 4<br>NO LIQUIDS .... 5<br>NOT SURE.................................... 8	
326	Was (NAME) given the same amount to eat, less or more than usual?<br><br>IF LESS, ASK: A little less or a lot less?	MUCH LESS................................. 1<br>A LITTLE LESS............................ 2<br>SAME AMOUNT........................... 3<br>MORE... 4<br>NO FOOD .... 5<br>NOT SURE.................................. 8	
327	Did you get medical advice or treatment for the illness?	YES……………………............ 1<br>NO…………………………………… 2	If 2, go to 330
328	Where did you seek medical advice or treatment?<br>Anywhere else?<br><br>NOTE ALL RESPONSES<br><br>______________________________<br>(NAME OF PLACE)	**PUBLIC SECTOR**<br>HEALTH FACILITY.................... A<br>MOBILE HEALTH BRIGADE…. B<br>OTHER PUBLIC________........ C<br>(SPECIFY)<br><br>**PRIVATE SECTOR**<br>CLINIC....................................... D<br>PHARMACY.............................. E<br>DOCTOR..................... ………. F<br>OTHER ________________...... G<br>(SPECIFY)<br><br>**OTHER LOCATION**<br>MARKET....................................... H<br>PRACTICIONER OF TRADITIONAL MEDICINE……….. I<br>NEIGHBORHOOD HEALTH PERSONELL………………………. J	

NO.	QUESTIONS	CODING CATEGORIES		SKIP
		OTHER ________________......... (SPECIFY)	X	
329	Where was the first place you sought treatment?<br>USE CATEGORIES FROM 328.	FIRST PLACE………………………	□	
330	During the time (NAME) was sick, was he/she given any medication?	YES…………………….................<br>NO……………………………………<br>NOT SURE…………………………	1<br>2<br>8	If 2 or 8, go to 294 of next column, or if there are no more, go to section 5
331	Which medications did (NAME) take?<br><br>NOTE ALL MEDICINES MENTIONED	**ANTIMALARIAL DRUGS**<br>FANSIDAR................................<br>CHLOROQUIN..........................<br>AMODIAQUINE.........................<br>QUININE..................................<br>ARTEMISININA.........................<br>COARTEM................................<br>OTHER ANTI-MALARIA ……….<br>___________________<br>(SPECIFY)<br><br>**ANTIBIOTIC**<br>PILLS/SYRUP…………………...<br>INJECTION………………………<br><br>**OTHER MEDICATIONS**<br>ASPIRIN ....<br>ACETAMINOPHEN...................<br>IBUPROFEN.............................<br>OTHER ____________............<br>(SPECIFY)<br>NOT SURE....................................	A<br>B<br>C<br>D<br>E<br>F<br>G<br><br><br><br><br>H<br>I<br><br><br>J<br>K<br>L<br>X<br><br>Z	
332	How long after the fever began was treatment started?	SAME DAY....................................<br>NEXT DAY....................................<br>TWO DAYS LATER.......................<br>THREE OR MORE DAYS LATER..<br>NOT SURE....................................	0<br>1<br>2<br>3<br>8	

SECTION 5 NON-TRANSMISSIBLE DISEASES - ADULTS

NO.	QUESTIONS	CODING CATEGORIES		SKIP
601	How often do you have a drink containing alcohol?	NEVER..<br>ONCE A MONTH OR LESS………<br>2 TO 4 TIMES A MONTH..............<br>2 TO 4 TIMES A WEEK…………..<br>4 OR MORE TIMES A WEEK…….	1<br>2<br>3<br>4<br>5	If 1, go to 611
602	Think of a day in which you drink alcohol.<br>How many standard drinks containing alcohol do you have on a typical day when drinking?<br><br>How many cans / bottles / glasses?	0 OR 1………………………………<br>2 OR 3………………………………<br>4 OR 5………………………………<br>6 OR 7………………………………<br>8 OR more…………………………..	1<br>2<br>3<br>4<br>5	
603	In the last year, how often did you have six or more drinks in one day?	NEVER..<br>LESS THAN ONCE A MONTH......<br>MONTHLY....................................<br>WEEKLY.......................................<br>EVERY DAY OR ALMOST<br>EVERY DAY.................................	1<br>2<br>3<br>4<br><br>5	
604	During the past year, how often have you found that you were not able to stop drinking once you had started?	NEVER..<br>LESS THAN ONCE A MONTH......<br>MONTHLY....................................<br>WEEKLY.......................................<br>EVERY DAY OR ALMOST<br>EVERY DAY.................................	1<br>2<br>3<br>4<br><br>5	

NO.	QUESTIONS	CODING CATEGORIES		SKIP
610	Has a relative or friend, doctor or other health worker been concerned about your drinking or suggested you cut down?	NO…………………………………..<br>YES, BUT NOT IN THE LAST 12 MONTHS......................................<br>YES, IN THE LAST 12 MONTHS..	1<br><br>2<br>3	
611	Do you currently smoke cigarettes?	YES……………………................<br>NO……………………………………	1<br>2	If 2, go to 614
612	How often do you smoke cigarettes?	DAILY..<br>WEEKLY.......................................<br>MONTHLY.....................................<br>LESS THAN ONCE A MONTH......<br>NEVER..	1<br>2<br>3<br>4<br>5	
613	In the last 24 hours, how many cigarettes did you smoke?	NUMBER OF CIGARETTES	□□	
614	Do you currently use any other type of tobacco?	YES……………………................<br>NO……………………………………	1<br>2	If no, go to 616
615	What sort of tobacco do you currently smoke or consume?<br><br>NOTE ALL TYPES MENTIONED	PIPE…………………………………<br>ROLLED CIGARETTES…………..<br>CIGAR………………………………<br>SNUFF………………………………<br>OTHER ________________.........<br>(SPECIFY)	A<br>B<br>C<br>D<br>X	
616	How often do you chew tobacco?	DAILY..<br>WEEKLY.......................................<br>MONTHLY.....................................<br>LESS THAN ONCE A MONTH......<br>NEVER..	1<br>2<br>3<br>4<br>5	
617	Have you ever had your blood pressure measured?	YES……………………................<br>NO……………………………………<br>NOT SURE…………………………	1<br>2<br>8	
618	Has a health care worker ever told you you have high blood pressure or hypertension?	YES……………………................<br>NO……………………………………<br>NOT SURE…………………………	1<br>2<br>8	
619	Are you currently receiving any of the following for your blood pressure?<br><br>INTERVIEWER: read all options and select all that apply.	MEDICATION................................<br>ADVICE TO CHANGE DIET………<br>ADVICE TO LOSE WEIGHT……...<br>ADVICE TO DO MORE EXERCISE…………………………<br>ADVICE OR TREATMENT TO GIVE UP SMOKING………………	1<br>2<br>3<br><br>4<br><br>5	
620	Has a health professional ever measured your blood sugar?	YES……………………................<br>NO……………………………………<br>NOT SURE…………………………	1<br>2<br>8	If 2 or 8, go to 623
621	Has a health professional ever told you have high blood sugar or diabetes?	YES……………………................<br>NO……………………………………	1<br>2	If 2, go to 623
622	Do you take any medication for diabetes?	YES……………………................<br>NO……………………………………	1<br>2	
623	Did you ever have a heart attack, chest pain due to heart disease or a stroke?	YES……………………................<br>NO……………………………………<br>NOT SURE…………………………	1<br>2<br>8	
624	Do you take any medication to prevent or treat cardiovascular disease?	YES……………………................<br>NO……………………………………	1<br>2	
625	**FOR WOMEN:**<br>Have you ever been screened for breast cancer?	YES……………………................<br>NO……………………………………<br>NOT SURE…………………………	1<br>2<br>8	
625A	What was the result of this screening?	NEGATIVE…………………………<br>POSITIVE…………………………..<br>NOT SURE…………………………	1<br>2<br>8	
626	**FOR WOMEN:**<br>Have you ever been screened for prevention or early detection of cervical cancer?	YES……………………................<br>NO……………………………………<br>NOT SURE…………………………	1<br>2<br>8	
626A	What was the result of this screening?	NEGATIVE…………………………<br>POSITIVE…………………………..<br>NOT SURE…………………………	1<br>2<br>8	
627	Have you heard of the HPV vaccine, that is, a vaccine to prevent cervical cancer?	YES……………………................<br>NO……………………………………<br>NOT SURE…………………………	1<br>2<br>8	
628	Have you ever received the HPV vaccine, that is, a vaccine to prevent cervical cancer?	YES……………………................<br>NO……………………………………<br>NOT SURE…………………………	1<br>2<br>8	

NO.	QUESTIONS	CODING CATEGORIES	SKIP
	Now I'd like to ask you a few questions about mental health.		
629	Have you ever sought care for a mental health problem?	YES……………………….............. 1<br>NO…………………………………… 2	
630	What was the mental health problem?	EPILEPSY………………………….. 1<br>DEMENTIA…………………………. 2<br>SCHIZOPHRENIA…………………. 3<br>DEPRESSION…………………….. 4	
631	Were you treated for this problem?	YES……………………….............. 1<br>NO…………………………………… 2	
632	Where were you treated?<br><br>*INTERVIEWER: SELECT ALL THAT APPLY*	CLINIC / HOSPITAL...................... 1<br>TRADITIONAL HEALER............... 2<br>FRIEND... 3<br>FAMILY MEMBER........................ 4<br>COMMUNITY GROUP.................. 5<br>OTHER ________________......... 6<br>(SPECIFY)	
633	How were you treated?<br><br>*INTERVIEWER: SELECT ALL THAT APPLY*	MEDICATION................................ 1<br>COUNSELING............................... 2<br>PHYSICAL EXAM.......................... 3<br>SURGERY..................................... 4<br>EMOTIONAL SUPPORT................ 5<br>PLANTS OR HERBS..................... 6<br>PRAYER.. 7<br>OTHER ________________......... 8<br>(SPECIFY)	
634	Have you ever had a period of sadness or loss of energy that lasted more than two weeks?	YES……………………….............. 1<br>NO…………………………………… 2	
635	Were you treated for this problem?	YES……………………….............. 1<br>NO…………………………………… 2	
636	Where were you treated?<br><br>*INTERVIEWER: SELECT ALL THAT APPLY*	CLINIC / HOSPITAL...................... 1<br>TRADITIONAL HEALER............... 2<br>FRIEND... 3<br>FAMILY MEMBER........................ 4<br>COMMUNITY GROUP.................. 5<br>OTHER ________________......... 6<br>(SPECIFY)	
637	How were you treated?<br><br>*INTERVIEWER: SELECT ALL THAT APPLY*	MEDICATION................................ 1<br>COUNSELING............................... 2<br>PHYSICAL EXAM.......................... 3<br>SURGERY..................................... 4<br>EMOTIONAL SUPPORT................ 5<br>PLANTS OR HERBS..................... 6<br>PRAYER.. 7<br>OTHER ________________......... 8<br>(SPECIFY)	
638	Have you ever in your life had thoughts of suicide or self-harm?	YES……………………….............. 1<br>NO…………………………………… 2	
639	In the last month, have you had thoughts of suicide or self-harm?	YES……………………….............. 1<br>NO…………………………………… 2	
640	Do you currently have thoughts of suicide or self-harm?	YES……………………….............. 1<br>NO…………………………………… 2	
641	Have you sought treatment for this?	YES……………………….............. 1<br>NO…………………………………… 2	
642	Where were you treated?<br><br>*INTERVIEWER: SELECT ALL THAT APPLY*	CLINIC / HOSPITAL...................... 1<br>TRADITIONAL HEALER............... 2<br>FRIEND... 3<br>FAMILY MEMBER........................ 4<br>COMMUNITY GROUP.................. 5<br>OTHER ________________......... 6<br>(SPECIFY)	
643	How were you treated?<br><br>*INTERVIEWER: SELECT ALL THAT APPLY*	MEDICATION................................ 1<br>COUNSELING............................... 2<br>PHYSICAL EXAM.......................... 3<br>SURGERY..................................... 4<br>EMOTIONAL SUPPORT................ 5<br>PLANTS OR HERBS..................... 6<br>PRAYER.. 7	

NO.	QUESTIONS	CODING CATEGORIES	SKIP
		OTHER ________________......... 8<br>(SPECIFY)	
	I'd like to ask you some questions about your perception of mental illness.		
644	Would you be willing to have a friend with mental illness?	DEFINITELY WILLING.................. 1<br>PROBABLY WILLING................... 2<br>PROBABLY UNWILLING.............. 3<br>DEFINITELY UNWILLING............. 4	
703	Would you be willing to let someone with mental illness take care of your children?	DEFINITELY WILLING.................. 1<br>PROBABLY WILLING................... 2<br>PROBABLY UNWILLING.............. 3<br>DEFINITELY UNWILLING............. 4	
645	Would you be willing to assist someone with mental illness?	DEFINITELY WILLING.................. 1<br>PROBABLY WILLING................... 2<br>PROBABLY UNWILLING.............. 3<br>DEFINITELY UNWILLING............. 4	
646	People with mental illness should be chained, tied and locked in their homes.	AGREE COMPLETELY................. 1<br>AGREE SOMEWHAT.................... 2<br>DISAGREE SOMEWHAT.............. 3<br>DISAGREE COMPLETELY........... 4	
647	It is possible to catch mental illness from treating or helping someone who is mentally ill.	AGREE COMPLETELY................. 1<br>AGREE SOMEWHAT.................... 2<br>DISAGREE SOMEWHAT.............. 3<br>DISAGREE COMPLETELY........... 4	
648	Anyone can have a mental illness or mental health problems.	AGREE COMPLETELY................. 1<br>AGREE SOMEWHAT.................... 2<br>DISAGREE SOMEWHAT.............. 3<br>DISAGREE COMPLETELY........... 4	
649	Mental health problems are caused by witchcraft or a curse placed on a person by someone else.	AGREE COMPLETELY................. 1<br>AGREE SOMEWHAT.................... 2<br>DISAGREE SOMEWHAT.............. 3<br>DISAGREE COMPLETELY........... 4	
	Now I would like to ask you questions about Epilepsy		
650	Did you or a child in the household ever have attacks of shaking of the arms or legs which you could not control?	YES, MYSELF……………………. 1<br>NO……………………………………. 2<br>CHILD UNDER 15 YEARS………. 3	
651	Have you or a child in the houshold ever had attacks in which you fall suddenly, without any reason, changing color in the palms, lips or face?	YES, MYSELF……………………. 1<br>NO……………………………………. 2<br>CHILD UNDER 15 YEARS………. 3	
652	Have you or a child in the houshold ever lost consciousness?	YES, MYSELF……………………. 1<br>NO……………………………………. 2<br>CHILD UNDER 15 YEARS………. 3	
653	Have you or a child in the houshold ever had attacks in which you fall with loss of consciousness?	YES, MYSELF……………………. 1<br>NO……………………………………. 2<br>CHILD UNDER 15 YEARS………. 3	
654	Have you or a child in the houshold ever had attacks in which you fall and bite your tongue?	YES, MYSELF……………………. 1<br>NO……………………………………. 2<br>CHILD UNDER 15 YEARS………. 3	
655	Have you or a child in the houshold ever had attacks in which you fall and lose control of your bladder?	YES, MYSELF……………………. 1<br>NO……………………………………. 2<br>CHILD UNDER 15 YEARS………. 3	
656	Have you or a child in the houshold ever had brief attacks of shaking or trembling in one arm or leg or in the face?	YES, MYSELF……………………. 1<br>NO……………………………………. 2<br>CHILD UNDER 15 YEARS………. 3	
657	Have you or a child in the houshold ever had attacks in which you lost contact with surroundings or experience abnormal smells?	YES, MYSELF……………………. 1<br>NO……………………………………. 2<br>CHILD UNDER 15 YEARS………. 3	
658	Have you or a child in the houshold ever been told that you have or have had convulsions, epilepsy or epileptic fits?	YES, MYSELF……………………. 1<br>NO……………………………………. 2<br>CHILD UNDER 15 YEARS………. 3	
	Now I would like to ask you about your overall health.		
659	How much difficulty do you have in standing for long periods of time (30 minutes)?	NONE... 1<br>LITTLE.. 2<br>SOME... 3<br>A LOT... 4<br>VERY MUCH OR UNABLE............ 5	

NO.	QUESTIONS	CODING CATEGORIES		SKIP
660	How much difficulty do you have in doing household chores?	NONE..<br>LITTLE...<br>SOME..<br>A LOT..<br>VERY MUCH OR UNABLE...........	1<br>2<br>3<br>4<br>5	
661	How much difficulty do you have in learning a new task, such as the route to a new place?	NONE..<br>LITTLE...<br>SOME..<br>A LOT..<br>VERY MUCH OR UNABLE...........	1<br>2<br>3<br>4<br>5	
662	How much difficulty do you have in participating in community activities (such as festivals, religious services or others) in the same way as other people?	NONE..<br>LITTLE...<br>SOME..<br>A LOT..<br>VERY MUCH OR UNABLE...........	1<br>2<br>3<br>4<br>5	
663	How does your health affect your emotional state?	NONE..<br>LITTLE...<br>SOME..<br>A LOT..<br>VERY MUCH OR UNABLE...........	1<br>2<br>3<br>4<br>5	
664	How much difficulty do you have in concentrating on a task for ten minutes?	NONE..<br>LITTLE...<br>SOME..<br>A LOT..<br>VERY MUCH OR UNABLE...........	1<br>2<br>3<br>4<br>5	
665	How much difficulty do you have in walking a long distance, e.g. one kilometer or the equivalent?	NONE..<br>LITTLE...<br>SOME..<br>A LOT..<br>VERY MUCH OR UNABLE...........	1<br>2<br>3<br>4<br>5	
666	How much difficulty do you have in taking a bath?	NONE..<br>LITTLE...<br>SOME..<br>A LOT..<br>VERY MUCH OR UNABLE...........	1<br>2<br>3<br>4<br>5	
667	How much difficulty do you have in dressing?	NONE..<br>LITTLE...<br>SOME..<br>A LOT..<br>VERY MUCH OR UNABLE...........	1<br>2<br>3<br>4<br>5	
668	How much difficulty do you have in dealing with strangers?	NONE..<br>LITTLE...<br>SOME..<br>A LOT..<br>VERY MUCH OR UNABLE...........	1<br>2<br>3<br>4<br>5	
669	How much difficulty do you have in maintaining friendships?	NONE..<br>LITTLE...<br>SOME..<br>A LOT..<br>VERY MUCH OR UNABLE...........	1<br>2<br>3<br>4<br>5	
670	How much difficulty do you have at work or in school day-to-day?	NONE..<br>LITTLE...<br>SOME..<br>A LOT..<br>VERY MUCH OR UNABLE...........	1<br>2<br>3<br>4<br>5	
671	The last time you went to a clinic or hospital, which one did you go to?	Never went for an outpatient visit...<br>Name of clinic................................	0<br>□	
672	The last time you went to an emergency room, which health center or hospital did you go to?	Never went for an outpatient visit...<br>Name of clinic	0<br>□	
